# Immunological and inflammatory responses in the kidneys in experimental acanthamoebiasis

**DOI:** 10.1128/spectrum.00243-25

**Published:** 2025-06-30

**Authors:** Karolina Kot, Patrycja Tomasiak, Maciej Tarnowski, Danuta Kosik-Bogacka, Natalia Łanocha-Arendarczyk

**Affiliations:** 1 Department of Biology, Parasitology, and Pharmaceutical Botany, Pomeranian Medical University in Szczecin 37805, Szczecin, Poland; 2 Institute of Physical Culture Sciences, University of Szczecin 49673, Szczecin, Poland; 3 Department of Physiology in Health Sciences, Pomeranian Medical University in Szczecin 37805, Szczecin, Poland; University of Edinburgh, Midlothian, United Kingdom

**Keywords:** *Acanthamoeba *sp., COX-2, cytokines, immunological status, kidneys, NLRP3

## Abstract

The pathomechanisms of *Acanthamoeba* spp. infection are still poorly understood. The aim of the study was to determine the expression of the NLR family pyrin domain-containing protein 3 (NLRP3), prostaglandin-endoperoxide synthase 2 (PTGS2, commonly known as cyclooxygenase-2, COX-2), and various cytokines in the kidneys of immunocompetent and immunosuppressed mice infected with *Acanthamoeba* sp. (T16 genotype). The proteins were analyzed by quantitative reverse transcription PCR. In immunocompetent mice, we observed increased mRNA levels of interferon gamma (IFNγ), tumor necrosis factor alpha (TNFα), PTGS2/COX-2, and interleukin (IL)-17A at the beginning of infection. While in the late stages, we found decreased levels of NLRP3, IL-1β, IL-10, IL-17A, IL-21, IL-23, IFNγ, TNFα, and macrophage inflammatory protein-2 (MIP-2) in the kidneys of infected hosts compared to uninfected ones. In immunosuppressed mice, we noted higher expressions of NLRP3, PTGS2/COX-2, IL-1β, IL-10, IL-17A, IL-21, IFNγ, TNFα, and MIP-2, and lower expression of IL-18 in the infected mice compared to the control group. The basic understanding of the immune response during *Acanthamoeba* sp. infection is critical to improving clinical outcomes and also has significant implications for developing therapeutic interventions. Here, we have reported the inflammatory responses in the kidneys infected with *Acanthamoeba* sp. However, additional studies are needed to understand the specific beneficial and detrimental roles of the cytokine response.

**IMPORTANCE:**

*Acanthamoeba* spp. are significant biological factors and can cause rare infections characterized by high mortality and difficulty with treatment. One of the reasons *Acanthamoeba* spp. persist so effectively in the body is a lack of knowledge about the pathomechanisms and pathophysiology of infections. Recent studies showed that *Acanthamoeba* spp. can also infect the kidneys in the hosts, being an important cause of medical complications. A better understanding of the mechanisms by which *Acanthamoeba* spp. cause kidney injury could lead to more effective treatments for systemic acanthamoebiasis.

## INTRODUCTION

Disseminated acanthamoebiasis is a multisystem disease caused by the opportunistic free-living amoebae, *Acanthamoeba* spp. The parasites can enter various organs, including the kidneys, by migrating with the blood from its initial location in the skin, subcutaneous tissue, or lungs (1). Most vulnerable to infection are people with impaired immunity, including transplant recipients and kidney failure patients (2–8). Renal involvement in acanthamoebiasis is mostly detected histologically in autopsies of patients with disseminated infection (9). The incidence of *Acanthamoeba* infections is low, but it is believed that many cases may be unrecognized (10). The pathomechanisms of amoebae infection remain incompletely understood, despite significant progress made in recent years. Moreover, there is no definitive and effective therapy for free-living amoebae infections, underscoring the critical importance of the immune system’s response (11, 12).

The inflammatory response is the immune response to infectious and non-infectious activators. The innate immune system recognizes invading microorganisms and danger signals in the body through specific pattern recognition receptors (PRRs). Currently known PRRs are divided into five types based on protein domain homology. The most extensively studied PRRs are Toll-like receptors (TLRs) located on the cell membrane and NOD-like receptors (NLRs) located in the cytoplasm (13). Stimulation of receptors initiates a signaling cascade that triggers immune response cells to produce pro-inflammatory cytokines (14). NLRP3 is the most comprehensive inflammasome in the family of NLRs. As an intracellular pattern recognition receptor, the NLRP3 inflammasome plays an important role in stimulating and regulating immune inflammation. Activation of the NLRP3 inflammasome is involved in acute and chronic kidney inflammation through induction of interleukin 1β (IL-1β) and IL-18 secretion leading to an autoimmune and inflammatory response (15, 16). Toll-like receptors are activated by interaction with their ligands, leading to the production of inflammatory cytokines, chemokines, and interferons. In addition, they are involved in the maturation and differentiation of antigen-presenting cells, thus linking the innate and acquired immune response (17). In studies of systemic acanthamoebiasis in a mouse model, there was increased expression of TLR2 in the kidneys of immunosuppressed hosts. In immunocompetent animals infected with *Acanthamoeba* sp., there were no statistically significant differences in TLR2 and TLR4 expressions compared to non-infected hosts (18). Research on the role of NLRs in *Acanthamoeba* sp. infection has not yet been conducted.

One of the significant components of the immune system is inflammation, as it is involved in protection against infection and repair of tissue damage. During the early phase of inflammation, it exerts cellular protection, and during late phases, it induces cellular damage. The immune system and inflammation play a role in the pathophysiology of many diseases (19). The host response to parasitic infection is regulated by the controlled production of small proteins called cytokines which modify and regulate immune and inflammatory responses (20). The primary role of cytokines is to modulate the proliferation of immunoregulatory cells in a pro-inflammatory or anti-inflammatory manner. Some investigators have postulated that the balance between the opposing actions of pro- and anti-inflammatory cytokines is what keeps tissues in a healthy and regulated state (19, 21). Cytokines can be divided into a number of families based on their structural similarities, including tumor necrosis factors (TNFs, e.g., TNFα), interferons (IFNs, e.g., IFNγ), and ILs (including IL-1β, IL-6, IL-17A, etc.). Secondly, they can also be classified according to their roles as pro-inflammatory or anti-inflammatory. Classically, IL-1β, IL-17, IL-21, TNFα, and interferons are considered pro-inflammatory. In contrast, IL-10 is described as anti-inflammatory. However, it should be noted that this classification is imperfect, as several cytokines (e.g., IL-6) can have both pro- and anti-inflammatory properties depending on the context (22, 23). The central link to various inflammatory processes is played by cyclooxygenase-2 (COX-2; also known as prostaglandin-endoperoxide synthase 2, PTGS2) (24). COX-2 is an inducible early response gene and is activated in response to various extracellular or intracellular stimuli, including various cytokines (25). The COX-2 expression in host cells has been reported in several different parasitic diseases, including those that have an affinity to kidneys (26).

The aim of the study was to analyze gene expressions of NLRP3, PTGS2/COX-2, IL-6, IL-1β, IL-10, IL-17A, IL-18, IL-21, IL-23, TNFα, IFNγ, and macrophage inflammatory protein-2 (MIP-2) in the kidneys of mice with systemic acanthamoebiasis. Since systemic infection primarily affects hosts with a reduced level of immune response, both animals with normal and reduced immune mechanisms were used in the study.

## RESULTS

### NLRP3

**Fig 1:**
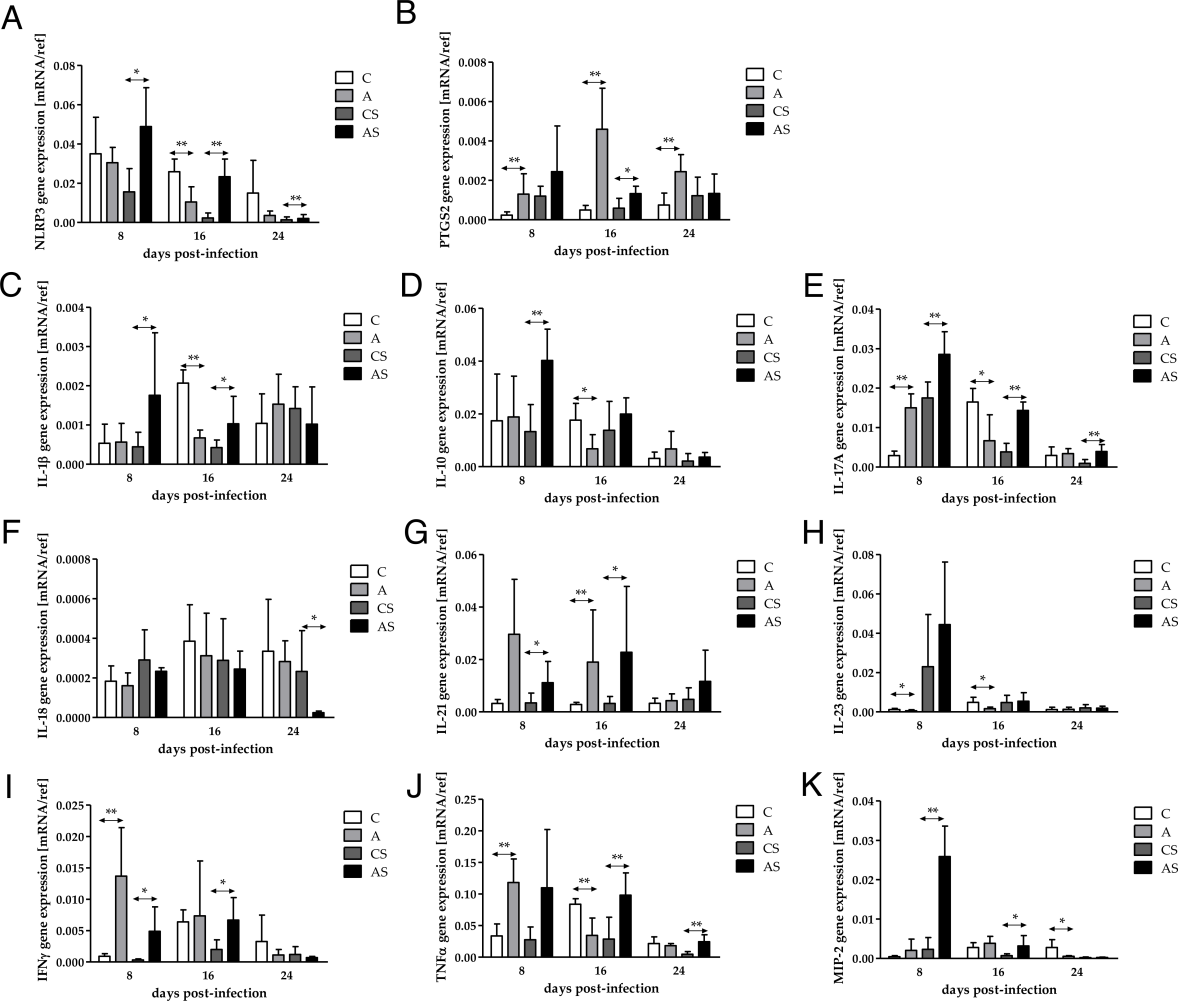
The gene expression of NLRP3 (**A**), PTGS2/COX2 (**B**), IL-1β (**C**), IL-10 (**D**), IL-17A (**E**), IL-18 (**F**), IL-21 (**G**), IL-23 (**H**), IFNγ (**I**), TNFα (**J**), and MIP-2 (**K**) in the kidneys of mice infected with *Acanthamoeba* sp. The mRNA expressions were determined using quantitative reverse transcription PCR. Data represent the means and standard deviations for six experiments. * indicates statistically significant differences *P* < 0.05, ** indicates statistically significant differences *P* < 0.01, Mann-Whitney U-test. A, immunocompetent mice infected with *Acanthamoeba* sp.; AS, immunosuppressed mice infected with *Acanthamoeba* sp.; C, immunocompetent uninfected mice; CS, immunosuppressed uninfected mice.

The mRNA level of NLRP3 in the immunocompetent mice decreased at each time point (H = 12.59, *P* = 0.002). Further analysis with Dunn’s test showed a difference only between 24 dpi and 8 dpi. A statistically significant difference between the A (immunocompetent mice infected with *Acanthamoeba* sp.) and C (immunocompetent control mice) groups was observed at 16 dpi (U = 1.00, *P* = 0.004; Fig. 1A); there was 38% lower gene expression in the A group.

Gene expression of NLRP3 in the immunosuppressed mice also decreased at each time point (H = 13.72, *P* = 0.001). Further analysis with Dunn’s test showed a difference only between 24 dpi and 8 dpi. There were differences between the AS (immunosuppressed mice infected with *Acanthamoeba* sp.) and CS (immunosuppressed control mice) groups at 8, 16, and 24 dpi; on each studied day, the mRNA level of NLRP3 in the AS was higher (8 dpi: U = 4.00, *P* = 0.03; 16 dpi: U = 0.00, *P* = 0.002; 24 dpi: U = 1.00, *P* = 0.004; Fig. 1A). Interestingly, the difference between the gene expression at 16 dpi was an order of magnitude.

We also observed the difference between the A and AS groups at 16 dpi. A higher gene expression was observed in the AS group (U = 5.00, *P* = 0.04).

### PTGS2/COX2

The mRNA level of PTGS2 in the immunocompetent mice changed during the course of infection (H = 9.51, *P* = 0.009). The analysis with Dunn’s test showed a difference between 16 dpi and 8 dpi. Additionally, there were statistically significant differences between the A and C groups at each time point (8 dpi: U = 1.00, *P* = 0.004; 16 dpi: U = 0.00, *P* = 0.002; 24 dpi: U = 1.00, *P* = 0.004; Fig. 1B); there was higher gene expression in the A group.

Gene expression of PTGS2 in the immunosuppressed mice did not change statistically at each time point. There was a statistically significant difference between the AS and CS groups at 16 dpi; the mRNA expression was two times higher in the AS (U = 4.00, *P* = 0.02; Fig. 1B).

We also observed the difference between the A and AS groups at 16 dpi as well as 24 dpi. A higher gene expression was observed in the AS group (16 dpi: U = 2.00, *P* = 0.008; 24 dpi: U = 5.00, *P* = 0.04).

### IL-6

Even though four different pairs of primers were used in the study, no expression was detected either in the kidneys of control mice or in the kidneys of infected mice. Some primers were designed by ourselves, and some were used from publications (27).

### IL-1β

The mRNA level of IL-1β in the immunocompetent mice increased at 16 dpi, and then decreased at 24 dpi (H = 10.84, *P* = 0.004). Analysis with Dunn’s test showed a difference between 16 dpi and 8 dpi. A statistically significant difference between the A and C groups was observed at 16 dpi (U = 0.00, *P* = 0.002; Fig. 1C); the gene expression was higher in the A group.

Gene expression of IL-1β in the immunosuppressed mice did not change significantly at any time point. There were higher mRNA levels of IL-1β in the AS group at 8 and 16 dpi compared to the CS group (8 dpi: U = 3.00, *P* = 0.02; 16 dpi: U = 5.00, *P* = 0.04; Fig. 1C).

We also observed the difference between the A and AS groups at 8 as well as 16 dpi. Higher gene expressions were observed in the A group (8 dpi: U = 3.00, *P* = 0.02; 16 dpi: U = 5.00, *P* = 0.04).

### IL-10

The mRNA level of IL-10 in the immunocompetent mice did not differ significantly. A statistically significant difference between the A and C groups was observed at 16 dpi (U = 2.00, *P* = 0.009; Fig. 1D); the gene expression was higher in the C group.

Gene expression of IL-10 in the immunosuppressed mice decreased at each time point (H = 14.38, *P* = 0.0008). The analysis with Dunn’s test showed a difference only between 24 dpi and 8 dpi. There was a higher mRNA level of IL-10 in the AS group at 8 dpi compared to the CS group (8 dpi: U = 1.00, *P* = 0.004; Fig. 1D).

We also observed the difference between the A and AS groups at 8 as well as 16 dpi. Higher gene expressions were observed in the AS group (8 dpi: U = 4.50, *P* = 0.03; 16 dpi: U = 2.00, *P* = 0.009).

### IL-17A

The mRNA level of IL-17A in the immunocompetent mice decreased at each time point (H = 8.52, *P* = 0.01). The analysis with Dunn’s test showed a difference only between 24 dpi and 8 dpi. Statistically significant differences between the A and C groups were observed at 8 (U = 0.00, *P* = 0.002) and 16 dpi (U = 4.00, *P* = 0.03; Fig. 1E); the gene expression was higher at 8 dpi and lower at 16 dpi in the A group.

Gene expression of IL-17A in the immunosuppressed mice also decreased at each time point (H = 15.20, *P* = 0.0005). The analysis with Dunn’s test showed a difference only between 24 dpi and 8 dpi. There were differences between the AS and CS groups at 8, 16, and 24 dpi; on each studied day, the mRNA level of IL-17A in the AS was higher (8 dpi: U = 1.00, *P* = 0.004; 16 dpi: U = 0.00, *P* = 0.002; 24 dpi: U = 1.00, *P* = 0.004; Fig. 1E). Interestingly, the differences between the gene expression at 16 and 24 dpi were an order of magnitude.

We also observed the difference between the A and AS groups at 8 dpi. A higher gene expression was observed in the AS group (U = 0.00, *P* = 0.002).

### IL-18

The mRNA level of IL-18 in the immunocompetent mice did not differ significantly. There were no differences between the A and C groups at various time points (Fig. 1F).

Gene expression of IL-18 in the immunosuppressed mice slightly increased at 16 dpi and then decreased at 24 dpi (H = 11.99, *P* = 0.0025). Further analysis with Dunn’s test showed a difference between 24 dpi and 8 dpi and between 24 dpi and 16 dpi. There was a difference only between the AS and CS groups at 24 dpi. Interestingly, the difference was an order of magnitude higher in the control mice than in the infected animals (U = 3.00, *P* = 0.02; Fig. 1F). We also observed the difference between the A and AS groups at 24 dpi. A higher gene expression was observed in the A group (U = 0.00, *P* = 0.002).

### IL-21

The mRNA level of IL-21 in the immunocompetent mice did not differ significantly between days post-infection. There was a higher IL-21 gene expression in the A group at 16 dpi compared to the C group (U = 0.00, *P* = 0.002; Fig. 1G).

Gene expression of IL-21 in the immunosuppressed mice did not differ significantly. There were higher IL-21 gene expressions in the AS group at 8 and 16 dpi than in the CS group (8 dpi: U = 4.00, *P* = 0.03; 16 dpi: U = 4.00, *P* = 0.03; Fig. 1G).

### IL-23

The mRNA level of IL-23 in the immunocompetent mice did not differ significantly. There were lower IL-23 gene expressions in the A group at 8 as well as 16 dpi compared to the C group (8 dpi: U = 5.00, *P* = 0.04; 16 dpi: U = 5.00, *P* = 0.04; Fig. 1H).

Gene expression of IL-23 in the immunosuppressed mice decreased at each time point (H = 12.85, *P* = 0.002). Further analysis with Dunn’s test showed a difference between 24 dpi compared to 8 dpi. There were no differences between the AS and CS groups at various time points (Fig. 1H).

We also observed higher gene expression of IL-23 in the AS group at 8 dpi compared to the A group (U = 0.00, *P* = 0.002).

### IFNγ

The mRNA level of IFNγ in the immunocompetent mice decreased at each time point (H = 6.75, *P* = 0.03). Further analysis with Dunn’s test showed a difference between 24 dpi compared to 8 dpi. A statistically significant difference between the A and C groups was observed at 8 dpi (U = 1.00, *P* = 0.004; Fig. 1I); there was 93% higher gene expression in the A group.

Gene expression of IFNγ in the immunosuppressed mice slightly increased at 16 dpi and then decreased at 24 dpi (H = 8.67, *P* = 0.013). Analysis with Dunn’s test showed a difference between 24 dpi compared to 16 dpi. There were differences between the AS and CS groups at 8 and 16 dpi; the mRNA level of IFNγ in the AS was higher (8 dpi: U = 4.00, *P* = 0.03; 16 dpi: U = 4.00, *P* = 0.03; Fig. 1I).

Taking into account the immunological status of the host, we noted higher mRNA level of IFNγ in the A group than in the AS group at 8 dpi (U = 4.00, *P* = 0.03).

### TNFα

The mRNA level of TNFα in the immunocompetent mice decreased at each time point (H = 10.58, *P* = 0.005). Further analysis with Dunn’s test showed differences between 24 dpi and 8 dpi and between 16 dpi and 8 dpi. Statistically significant differences between the A and C groups were observed at 8 (U = 0.00, *P* = 0.002) and 16 dpi (U = 2.00, *P* = 0.008; Fig. 1J); the gene expression was higher at 8 dpi and lower at 16 dpi in the A group.

Gene expression of TNFα in the immunosuppressed mice did not change significantly between days post-infection. There were differences between the AS and CS groups at 16 and 24 dpi; the mRNA levels of TNFα in the AS were higher (16 dpi: U = 2.00, *P* = 0.009; 24 dpi: U = 0.00, *P* = 0.002; Fig. 1J).

We also observed the difference between the A and AS groups at 16 dpi. A higher gene expression was observed in the AS group (U = 2.00, *P* = 0.009).

### MIP-2

The mRNA level of MIP-2 in the immunocompetent mice did not differ significantly between days post-infection. Statistically significant differences between the A and C groups were observed in long-lasting infection (24 dpi: U = 5.00, *P* = 0.04; Fig. 1K).

Gene expression of MIP-2 in the immunosuppressed mice decreased at each time point (H = 15.20, *P* = 0.0005). Further analysis with Dunn’s test showed a difference between 24 dpi and 8 dpi. There were also differences between the AS and CS groups at 8 and 16 dpi; the mRNA levels of MIP-2 in the AS were higher (8 dpi: U = 0.00, *P* = 0.002; 16 dpi: U = 3.50, *P* = 0.02; Fig. 1K).

We noted the differences between the A and AS groups at 8 as well as 24 dpi. A higher gene expression was observed in the AS group at 8 dpi (U = 0.00, *P* = 0.002) and in the A group at 24 dpi (U = 2.00, *P* = 0.009).

### Principal component analysis (PCA)

**Fig 2:**
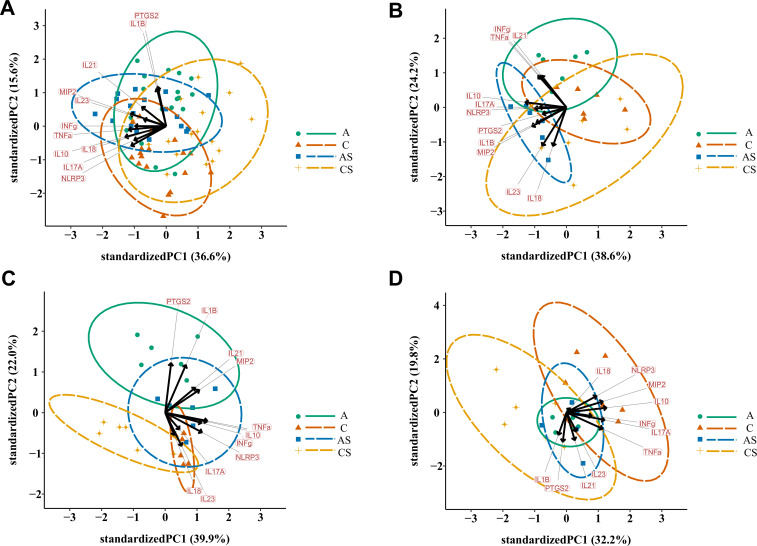
PCA of NLRP3, PTGS2, cytokines, and chemokine gene expression in the kidneys of mice infected with *Acanthamoeba* sp. Scatterplots of principal components 1 (PC1) and 2 (PC2) from all days post-infection, dpi (**A**) as well as only from 8 dpi (**B**), 16 dpi (**C**), and 24 dpi (**D**). Arrows show gene vectors, and each point represents an individual animal, with ellipses representing 95% confidence intervals around the means for each infection group. A, immunocompetent mice infected with *Acanthamoeba* sp.; AS, immunosuppressed mice infected with *Acanthamoeba*sp.; C, immunocompetent uninfected mice; CS, immunosuppressed uninfected mice.

To further assess possible changes in cytokine profile, and NLRP3 as well as PTGS2 gene expressions, between *Acanthamoeba* sp.-infected and uninfected animals, PCA was performed. The analysis showed that 52.2% of the total variance was accounted for by the first two principal components, and these were plotted. The biplot (across all dpi) did not show much clustering except that the C group was shifted to the bottom left of the graph—influenced more by principal component 2 (PC2) (Fig. 2A). The genes most affecting PC2 were PTGS2 and IL-1β, together contributing 69.5% of this component. However, it was decided to analyze the dpi separately, as shown in Fig. 2B through D.

At dpi 8 (Fig. 2B), PCA showed that 62.9% of the variation was accounted for by the first two principal components. The plot of PC2 versus PC1 did not show any separation between the ellipses. However, there appeared to be some divergence between mice belonging to the C and AS groups, mostly in the PC1 direction influenced by IL-10, MIP-2, NLRP3, and IL-1β contributing together 53.1% of PC1 variation, and between A and CS groups, mostly in the PC2 direction influenced by IL-18, IL-23, TNFα, IL-21, and IFNγ contributing together 89.9% of PC2 variation.

At dpi 16 (Fig. 2C), PCA showed that 62.0% of the variation was accounted for by the first two principal components, and only these were evaluated. The plot of PC2 versus PC1 showed that separation had occurred between the A and C groups, mostly in the PC2 direction influenced by PTGS2, IL-1β, and IL-23 contributing together 69.4% of PC2 variation, with some indication of divergence between infected and uninfected immunosuppressed mice, mostly in the PC1 direction influenced by TNFα, IL-10, NLRP3, IFNγ, and MIP-2 contributing together 79.3% of PC1 variation.

At dpi 24 (Fig. 2D), it showed that 52.0% of the variation was accounted for by the first two principal components, and only these were evaluated. The plot of PC2 versus PC1 showed no separation between ellipses, with some indication of divergence between the C and CS groups remaining, mostly in the PC1 direction influenced by IL-10, IL-17A, MIP-2, TNFα, and NLRP3 contributing together 87.9% of PC1 variation.

## DISCUSSION

Infectious diseases caused by different kinds of pathogenic organisms can result in tissue damage due to diverse pathogen virulence and dysregulated host responses. The immunological mechanisms of acanthamoebiasis are complex. Although multiple factors contribute to disease pathogenesis, it is primarily determined by the efficacy and quality of the host immune response. It is possible that the elimination of *Acanthamoeba* sp. requires the induction of cytokines and secretion of cytotoxic molecules, which leads to severe inflammation.

Cyclooxygenases are enzymes in the process of prostanoid synthesis. The existence of at least three isoenzymes has been proven. There are three forms: constitutive (COX-1), stress-inducible (COX-2), and located in the central nervous system (COX-3) (28). PTGS2/COX2 is tightly regulated, and its expression and activation are directly induced by pro-inflammatory cytokines and growth factors that activate intracellular inflammation-related pathways (29). In the lungs of mice from the same experimental model, the expression of COX-2 protein was higher in immunocompetent mice compared to controls. In immunosuppressed mice, the protein expression was at a similar level in infected and uninfected hosts (30). While in the eyes, COX-2-mediated production of prostaglandin E2 (PGE2) was similar in immunocompetent infected and uninfected hosts, while in the immunosuppressed animals, there was a lower level at 8 dpi and a higher level at 16 dpi in the infected than in the control group (31). In the kidneys of mice infected with *Acanthamoeba* sp., PTGS2/COX2 gene expression was higher in the A group at each time point compared to control mice. In the AS group, we observed higher expression only at 16 dpi. The delayed increase in COX-2 may be due to the effect of glucocorticosteroids. It was reported that methylprednisolone, which was used to reduce immunity in the AS group, suppresses the synthesis of COX-2 (32). Since COX-2 is involved in renal hemodynamics, sodium and water reabsorption, and blood pressure regulation (33), its increased expression in the kidneys caused by *Acanthamoeba* sp. may have implications for renal function.

The role of NLRP3 in regulating immune responses against most unicellular eukaryotic microorganisms is only beginning to be understood (34, 35). NLRP3 inflammasome is directly involved in modulating immune responses against *Leishmania* infection. It is reported that mice deficient in components of the NLRP3 inflammasome have increased susceptibility to *L. amazonensis* infection and defects in the clearance of the parasite (36). The NLRP3 inflammasome controls *Trypanosoma cruzi* parasitemia by inducing nitric oxide production via a caspase-1-dependent, IL-1 receptor-independent pathway (37). In malaria, on the other hand, NLRP3 and its associated cytokines have an immunopathologic role. Mice deficient in NLRP3 or IL-1β demonstrate significant resistance to *Plasmodium* infection when compared to wild-type mice (38, 39). In toxoplasmosis, NLRP3 is crucial for regulating the *Toxoplasma gondii* burden, controlling parasite growth and proliferation, and managing parasite-induced cell death. Additionally, it is suggested that *Toxoplasma gondii* may activate the inflammasome, and therefore, the parasite might manipulate inflammasome activation and inhibition, influencing immune responses and inflammation (40, 41). Other protozoa, such as *Entamoeba histolytica* and *Naegleria fowleri*, have been studied for the inflammatory response, including NLRP family and IL-1β production. *Entamoeba histolytica* activates the NLRP3 inflammasome and subsequently leads to IL-1β and IL-18 production (42). In THP-1 cells cocultured with *N. fowleri* trophozoites in the noncontact culture, the activation of caspase-1 and the production of IL-1β increased in a time-dependent manner, as did the formation and activation of the NLRP3 inflammasome (43). In immunocompetent hosts infected with *Acanthamoeba* sp., the parasite does not activate NLRP3, but in the immunosuppressed hosts, amoeba activates NLRP3 and subsequently induces the production of IL-1β. NLRP3 inflammasome and IL-1β play an important role in inducing renal inflammation and fibrosis (44, 45). This corresponds with our previous study in which we analyzed collagen deposition in the kidneys infected with *Acanthamoeba* sp. We found a higher percentage of collagen fibers in the A and AS groups compared to control groups at 24 dpi (46). In our hypothetical pathway, NLRP3 activation in host renal cells due to *Acanthamoeba* sp. infection is triggered through reactive oxygen species (ROS) and NADPH oxidase (NOX), similar to *Naegleria fowleri* (43). Bauernfeind et al. (47) revealed that NOXs may play a role in inducing ROS that is required to prime the NLRP3 inflammasome. In the *Acanthamoeba* sp.-infected kidneys, increased levels of NOX2 and NOX4 were noted (48). However, further studies are required to confirm our hypothetical inflammasome pathway.

IFNγ and TNFα are key cytokines in the Th1 immune response, crucial for Th1-type reactions (49). Th1 responses, involving macrophages and natural killer cells, are primarily responsible for direct pathogen attacks. Activated macrophages enhance pathogen phagocytosis and elimination efficacy. As the infection progresses, the immune response shifts from a Th1 to a Th2 type. In visceral leishmaniasis, the Th1 response is known to protect from infection, whereas the Th2 immune response is responsible for parasite growth and disease progression (50). In *Naegleria fowleri* infection, it is suggested that the amoeba manipulates the host immune response by transitioning from Th1 to Th2, thereby diminishing IFNγ production and attenuating phagocytosis and anti-amoebic activities, thus reducing the risk of the amoeba being targeted (51). In our previous study, we found that *Acanthamoeba* sp. induced a selective Th1 and Th2 response in immunocompetent hosts. In the case of hosts with low immunity, *Acanthamoeba* sp. elicited robust Th1 cell-mediated immunity (52). In the kidneys of immunocompetent *Acanthamoeba* sp.-infected mice, IFNγ and TNFα were increased only at the beginning of infection, while in the immunosuppressed mice, those cytokines were increased throughout the entire period of infection. IL-10, a regulatory cytokine produced by Th2 cells, was lower in the A group at 16 dpi and higher in the AS group but only at the beginning of infection. Failure to shift to a Th2 response might lead to continued Th1-mediated inflammation and tissue damage (51). This statement is confirmed by histopathological examination of the kidneys described in our previous paper (53). No pathomorphological changes were observed in the renal cortex, parenchyma of the medulla, or renal pelvis in the immunocompetent *Acanthamoeba* sp.-infected mice, but on the last day of infection, we found higher collagen deposition. In the immunosuppressed mice at 16 and 24 days post-*Acanthamoeba* sp. infection, poorly visible Bowman’s capsules and lighter staining of the nuclei as well as cytoplasm of tubular cells were observed. Additionally, amoeba-like cells were observed in mice from the AS group at 16 and 24 dpi, and higher collagen deposition was observed in the kidneys on the last day of infection (46, 53).

In response to tissue injury, IL-23 is produced by tissue-resident myeloid cells and promotes the expansion of Th17 cells, which produce and secrete IL-17A and other inflammatory mediators (54). IL-23 and IL-17 have been confirmed to markedly affect chronic inflammation. In addition, the discovery of the IL-23/IL-17 pathway has contributed to a clearer understanding of the underlying mechanism of inflammatory diseases (55). In some parasitic diseases, the role of IL-23 and IL-17 has been described. In malaria, for example, IL-23 and IL-17 reduce the severity of infection with blood-stage parasites (56). In the kidneys of immunocompetent and immunosuppressed mice with experimental acanthamoebiasis, there was no increased IL-23 gene expression, indicating no role of this cytokine in the immunological response to *Acanthamoeba* infection. IL-17A is a pro-inflammatory cytokine that plays a critical role in the migration and activation of inflammatory cells at the site of inflammation (57). Suryawanshi et al. (58) suggested that IL-17A production after *Acanthamoeba* spp. infection plays an important role in host protection against invading parasites in the human cornea during *Acanthamoeba* keratitis. A protective role of IL-17A in host defense against some parasites, including *Trypanosoma cruzi*, *Leishmania* sp., and *Toxoplasma gondii*, has also been documented (59–61). However, there is also some evidence showing the pathological role of IL-17 during parasitic infection (62). Overproduction of IL-17 promotes inflammation and autoimmunity. IL-17 recruits and stimulates different cells to drive chronic inflammation (63, 64). Additionally, IL-17A was found to induce expression and activity of extracellular matrix metalloproteinases 2 and 9 (MMP-2 and MMP-9, respectively) responsible for, e.g., extracellular matrix destruction and tissue damage (65, 66). In our previous study, we observed increased expression of IL-17A after incubation of the lymphocytes from the spleen of immunocompetent and immunosuppressed *Acanthamoeba* spp.-infected mice with increasing doses of concanavalin A (52). In the kidneys from the same murine model, we also found increased IL-17A gene expression in the A and AS groups. Additionally, we observed higher activity of MMP-2 and MMP-9 in the same days as IL-17A (67). It is difficult to conclude whether IL-17A has a protective or pathological role in the kidneys infected with *Acanthamoeba* spp., but the results rather tend to support a pathological effect of this cytokine.

IL-21 has been implicated in the regulation of Th1, Th2, Th17, and regulatory immune responses. This cytokine plays a significant role in T cell killing ability, regulating antibody production, and inflammatory responses (68, 69). It was reported that IL-21 is highly expressed in parasitized organs of infected humans as well as in murine models of human parasitic diseases (70). Capewell et al. (71) reported that IL-21 is a cause of the severity of human African trypanosomiasis, while Stumhofer et al. (68) found that IL-21 plays a key role in shaping the humoral and cellular response to *Toxoplasma gondii*, but they also indicated that IL-21 has a limited role in regulating immunopathology. In the kidneys infected with *Acanthamoeba* sp., we noted higher IL-21 gene expression in the immunocompetent hosts at 16 dpi and in the immunosuppressed host at 8 and 16 dpi. The role of this cytokine in immunological response to *Acanthamoeba* infection remains unknown.

MIP-2, a CXC chemokine, exerts effects on a number of cell types including neutrophils and induces the migration of neutrophils to sites of infection or tissue injury (72). MIP-2 may be responsible for the recruitment and activation of inflammatory cells during parasitosis (73). Infection with *Leishmania major* and *Plasmodium* spp. causes increased secretion of MIP-2 (74–77). Extracellular vesicles of *Naegleria fowleri* induced an increased expression of MIP-2 in BV-2 microglial cells (78). In our study concerning other free-living amoebae, *Acanthamoeba* sp., we observed lower gene expression in immunocompetent hosts with long-lasting acanthamoebiasis, and higher levels at 8 and 16 dpi in the immunosuppressed animals. *Acanthamoeba* sp. could promote the recruitment of neutrophils, which may exacerbate deleterious inflammation (79).

The basic understanding of the immune response during *Acanthamoeba* sp. infection is critical to improving clinical outcomes and also has significant implications for developing therapeutic interventions. Here, we have reported the immunological and inflammatory responses in the kidneys infected with *Acanthamoeba* sp. However, additional studies are needed to understand the specific beneficial and detrimental roles of the cytokine response. Studies of genetically deficient animals or with antibody blockade will be crucial to dissecting each cytokine’s role in the immune response and immunopathology during *Acanthamoeba* sp. infection. Designing therapeutics with the selective ability to inhibit or regulate the interaction between these molecules and renal cells could minimize host kidney damage.

## MATERIAL AND METHODS

### Animals

Six to 10-week-old male BALB/c mice (*n* = 96; mean weight 23 g) were obtained from the Centre of Experimental Medicine, Medical University in Bialystok, Poland, and maintained at a controlled temperature as well as with food and water *ad libitum*.

### Parasites

*Acanthamoeba* sp. (AM22 strain, T16 genotype, GenBank: GQ342607.1) was isolated from the bronchoaspirate of a hemato-oncology patient (80). The amoebae were maintained by serial *in vitro* passages on non-nutrient agar plates overlaid with deactivated *Escherichia coli* at 37°C.

### Experimental design

Mice were randomly divided into four groups:

- A, infected with *Acanthamoeba* sp., immunocompetent (*n* = 30)
- AS, infected with *Acanthamoeba* sp., immunosuppressed (*n* = 30)
- C, uninfected, immunocompetent (*n* = 18)
- CS, uninfected, immunosuppressed (*n* = 18).

Animals of the AS and CS groups were intraperitoneally injected with 220 µL of methylprednisolone sodium succinate (Solu-Medrol; Pfizer, New York, NY, USA) to suppress their immune response. Animals of the A and AS groups were intranasally infected with 10,000–20,000 trophozoites of *Acanthamoeba* sp., and animals of the C and CS groups received 3 µL of saline. Ten animals from the A and AS groups and six animals from the C and CS groups were euthanized by intraperitoneal administration of sodium pentobarbital (Euthasol vet, Raamsdonksveer, The Netherlands; 2 mL/kg body weight) prior to the removal of the kidneys. Organs were obtained at three moments post-infection: day 8 (early phase), day 16, and day 24 (late phase). Immediately after euthanasia, the kidneys were removed, immediately frozen, and stored at −80°C. The detailed experimental design is presented in the works of Łanocha-Arendarczyk et al. (30) and Kot et al. (46, 48, 67).

### Gene expression

The frozen kidneys of mice were homogenized, and then total RNA was isolated from them using RNeasy Mini Kit (Qiagen, Hilden, Germany) according to the manufacturer’s instructions. The concentration and purity of the isolated RNA were determined using a Nanodrop ND-1000 spectrophotometer (Thermo Fisher Scientific Co., Waltham, MA, USA).

Quantitative mRNA expressions of studied protein genes were performed by two-step quantitative real-time PCR. The gene expression was determined in relation to the average expression of housekeeping genes, *Gapdh* and *Beta-2 microglobulin*. Tissue-isolated RNA (1 µg) was prepared for analysis using a FirstStrand cDNA synthesis kit (Thermo Fisher ScientificTM, Waltham, MA, USA; cat no. K1612) and oligo-dT primers (Table 1; Warsaw, Poland). Real-time quantification of mRNA levels was performed using the 7500 Fast Real-Time PCR System (Applied Biosystems, Foster City, CA, USA) with Power SYBR Green PCR Master Mix (Applied Biosystems, Foster City, CA, USA, cat no. A25918). The analysis was repeated twice for each sample, and the arithmetic mean was calculated.

**TABLE 1:** Primers used in quantitative reverse transcription PCR

Gene	Forward	Reverse
*Gapdh*	GGA GAA ACC TGC CAA GTA TGA TG	GAC AAC CTG GTC CTC AGT GTA GC
*Beta-2 microglobulin*	CAT ACG CCT GCA GAG TTA AGC A	GAT CAC ATG TCT CGA TCC CAG TAG
*IL-1β*	TGC CAC CTT TTG ACA GTG ATG	AAG GTC CAC GGG AAA GAC AC
*IL-6*	GCC TTC TTG GGA CTG ATG CT	TGT GAC TCC AGC TTA TCT CTT GG
	TGA ACA ACG ATG ATG CAC TTG	CTG AAG GAC TCT GGC TTT GTC
	CTG CAA GAG ACT TCC ATC CA	AGT GGT ATA GAC AGG TCT GTT
	CTG CAA GAG ACT TCC AGC CA	AGT GGT ATA TAC TGG TCT GTT
*IL-10*	CTT ACT GAC TGG CAT GAG GAT	GCA GCT CTA GGA GCA TGT GG
*IL-17A*	TCA GCG TCT CCA AAC ACT GA	CGC CAA GGG AGT TAA AGA CT
*IL-18*	GAC TCT TGC GTC AAC TTC AAG G	CAG GCT GTC TTT TGT CAA CGA
*IL-21*	GGA CCC TTG TCT GTC TGG TA	TGT GGA GCT GAT ACA AGT TC
*IL-23*	GCT GGA TTG CAG AGC AGT AAT A	GCA TGC AGA GAT TCC GAG AGA G
*IFNγ*	CTC AAG TGG CAT AGA TGT GGA AG	GAT GGC CTG ATT GTC TTT CAA G
*MIP-2*	CGC TGT CAA TGC CTG AAG AC	ACA CTC AAG CTC TGG ATG TTC TTG
*NLRP3*	TGC GAT TCC GCT ATA AAT GCG	ACA AGT TCA TGT GGA TGA GG
*PTGS2/COX2*	AGG ACT GGG CCA TGG AGT	ACC TCT CCA CCA ATG ACC TG
*TNFα*	ACC GCC TGG AGT TCT GGA A	TGA TCC GCG ACG TGG AA

### Statistical analysis

Initially statistical analyses were performed using StatSoft Statistica 8.0 and GraphPad 4.0. For each of the studied parameters, the arithmetic mean and the standard deviation from the arithmetic mean (SD) were calculated. Due to the fact that the data did not follow normal distributions (according to the Shapiro-Wilk test), differences between the studied parameters were calculated by using the non-parametric Mann-Whitney U-tests (U) and Kruskal-Wallis tests (H) followed by Dunn’s *post-hoc* tests. A *P* -value of less than 0.05 was considered statistically significant.

Further multivariate analyses were performed using generalized least squares (GLS) and multivariate analysis of variance (MANOVA). All multivariate analyses used the R statistical platform (version 4.4.2; [81]); graphics were produced using R packages ggplot2 (82) and ggbiplot (83); but ellipses were drawn using the t distribution with 95% confidence. The mRNA expression data of the 11 test genes varied by three orders of magnitude. Therefore, after transformation using natural logarithm, these data were made relative to uninfected immunocompetent data for the same gene, log-fold datapoint = *log_e_* X_i,g_ − *mean*_j:n_(*log_e_* X_j,g,dpi8,con_) where X_i or j_ is an mRNA datapoint, *e* is Euler’s number, g is a gene, dpi8 is 8 days post-infection and con is immunocompetent uninfected (a difference of logged data is the same as taking a ratio for non-logged data).

Residuals and residual versus fitted plots were made for the model lm (log-fold data ~ dpi), as well as quantile-quantile plots, and Levene’s test for homogeneity of variance was performed.

To further counter heteroscedasticity, GLS regression with a weighted variance structure was performed. After weighting, the transformed errors will have constant variance. As the interaction between dpi and group was highly significant, the GLS used a per-dpi/group interaction variance structure. The R code was nlme::gls(“log-fold data” ~ dpi * group, weights = nlme::varIdent(~ 1 | interaction(dpi, group))) (84).

A multivariate analysis of variance was performed (R function: stats::manova) for log-fold data versus dpi + group + interaction, using a Pillai omnibus test (summary(manova_result, test = “Pillai”)). Omnibus tests for each gene were performed with dpi averaged over all four groups and infection group (A, AS, C, CS) averaged over all dpi, using Wald type III tests with the GLS heteroscedastic standard errors (R function: emmeans::joint_tests). *P*-values were adjusted for false discovery rate using the Benjamini-Hochberg method. Similar omnibus tests over all groups (for each dpi) were performed for each gene. The results of individual tests are presented in supplementary material (File S1 and S2).

PCAs were performed to indicate the relative effects of the 11 genes. (R code: stats::prcomp(gene_mat, center = TRUE, scale. = TRUE)). This was performed across all dpi and separately for dpi 8, dpi 16, and dpi 24.

## SUPPLEMENTAL MATERIAL

Supplemental File S1 spectrum.00243-25-s0001.docx Results of PCA.

Supplemental File S2 spectrum.00243-25-s0002.docx Code for PCA in the R statistical platform.

ASM does not own the copyrights to Supplemental Material that may be linked to, or accessed through, an article. The authors have granted ASM a non-exclusive, world-wide license to publish the Supplemental Material files. Please contact the corresponding author directly for reuse.
