# Supplementary material for: Immunological and inflammatory responses in the kidneys in experimental acanthamoebiasis
Source: Microbiol Spectr. 2025 Jun 30;13(8):e00243-25. doi: 10.1128/spectrum.00243-25 (PMC12323576; doi:10.1128/spectrum.00243-25)
Supplement: Supplemental File S1 — Results of PCA. [file spectrum.00243-25-s0001.docx]

**Results of PCA**

The mRNA expression (relative to house-keeping genes) data of the eleven test genes varied by three orders of magnitude (Fig. 1). Therefore, relative log-fold data were produced, which gave reasonable residual plots (Fig. S1), residual versus fitted (Fig. S2) and quantile-quantile plots (Fig. S3) showing that multivariate analysis could proceed.

**Fig. S1**. Time-dependent residuals for each gene (model lm(log-fold data ~ dpi) according to days post infection (dpi). Datapoints were logged and relative to mean logged values of immunocompetent uninfected mice at dpi 8.

**Fig. S2**. Residuals versus fitted values for each gene (model lm(log-fold data ~ dpi). Datapoints were logged and relative to mean logged values of immunocompetent uninfected mice at dpi 8.

**Fig. S3**. Quantile-quantile plots for each gene mRNA log-fold data. Datapoints were logged and relative to mean logged values of immunocompetent uninfected mice at dpi 8.

However, Levene's test for homogeneity of variance (for residuals versus dpi) still showed statistical significance (df = 2, F = 7.66, p = 0.0005), so it was decided to use generalized least squares with a weighted variance structure. The MANOVA model for log-fold data versus dpi + group + interaction, using a Pillai omnibus test, showed that group and dpi were highly significant (Table S1).

**Table S1.** Results of multivariate analysis of variance with a Pillai omnibus test for all log-fold data (model: log-fold data ~ dpi * group).

Df Pillai approx F num Df den Df Pr(>F)

dpi 2 1.0850 5.4975 22 102 1.134e-09 ***

group 3 1.9400 8.6524 33 156 < 2.2e-16 ***

dpi:group 6 2.2275 2.9522 66 330 9.830e-11 ***

Residuals 60

---

Signif. codes: 0 ‘***’ 0.001 ‘**’ 0.01 ‘*’ 0.05 ‘.’ 0.1 ‘ ’ 1

As the interaction was also highly significant, the subsequent GLS used a per-dpi/group interaction variance structure. Omnibus tests were performed with dpi averaged over all four groups and infection group (A, AS, C, CS) averaged over all dpi (Table S2).

**Table S2.** Results of generalized least squares regression (model: gls("log-fold data" ~ dpi * group with weights according to interaction between dpi and group). Non-statistically significant contrasts are shown in blue italics.

gene effect df1 df2 F p.value p_adj

1 NLRP3 dpi 2 60 18.407 5.839900e-07 1.167980e-06

2 NLRP3 group 3 60 12.034 2.869012e-06 2.869012e-06

*3 PTGS2 dpi 2 60 1.596 2.112517e-01 2.112517e-01*

4 PTGS2 group 3 60 25.243 1.094935e-10 2.189871e-10

5 IL_1B dpi 2 60 6.093 3.898068e-03 5.637609e-03

6 IL_1B group 3 60 4.624 5.637609e-03 5.637609e-03

7 IL_10 dpi 2 60 15.351 4.130925e-06 4.130925e-06

8 IL_10 group 3 60 15.154 1.880261e-07 3.760522e-07

9 IL_17A dpi 2 60 4.231 1.909793e-02 1.909793e-02

10 IL_17A group 3 60 5.113 3.236350e-03 6.472699e-03

11 IL_18 dpi 2 60 7.062 1.760606e-03 2.060129e-03

12 IL_18 group 3 60 5.516 2.060129e-03 2.060129e-03

*13 IL_21 dpi 2 60 0.944 3.947780e-01 3.947780e-01*

14 IL_21 group 3 60 10.968 7.706070e-06 1.541214e-05

15 IL_23 dpi 2 60 7.606 1.137246e-03 1.137246e-03

16 IL_23 group 3 60 13.600 7.092891e-07 1.418578e-06

17 INFgamma dpi 2 60 10.759 1.016135e-04 2.032270e-04

18 INFgamma group 3 60 5.748 1.592707e-03 1.592707e-03

19 TNFa dpi 2 60 3.206 4.754689e-02 4.754689e-02

20 TNFa group 3 60 13.356 8.777638e-07 1.755528e-06

21 MIP_2 dpi 2 60 13.389 1.556979e-05 3.113958e-05

22 MIP_2 group 3 60 8.399 9.533784e-05 9.533784e-05

This showed that all genes had statistically significant effects across groups, and all genes apart from PTGS2 and IL-21 had statistically significant effects across dpi. Therefore, no genes were removed from further analyses. Similar omnibus tests for each gene over all groups (for each dpi) were performed (Table S3).

**Table S3.** Results of generalized least squares regression (model: gls("log-fold data" ~ dpi * group with weights according to interaction between dpi and group). Statistically significant contrasts are shown in red italics.

gene by F df1 df2 p.value test p_adj

*1 NLRP3 com_inf 6.556 2 60 2.659436e-03 dpi_within_group 6.205350e-03*

2 NLRP3 com_uninf 0.537 2 60 5.872395e-01 dpi_within_group 5.872395e-01

*3 NLRP3 sup_inf 6.060 2 60 4.006151e-03 dpi_within_group 7.010764e-03*

*4 NLRP3 sup_uninf 13.769 2 60 1.198348e-05 dpi_within_group 4.194216e-05*

*5 PTGS2 com_inf 6.984 2 60 1.875670e-03 dpi_within_group 3.603387e-03*

*6 PTGS2 com_uninf 3.638 2 60 3.226655e-02 dpi_within_group 4.517317e-02*

7 PTGS2 sup_inf 0.711 2 60 4.954118e-01 dpi_within_group 4.954118e-01

8 PTGS2 sup_uninf 2.893 2 60 6.319565e-02 dpi_within_group 7.372825e-02

*9 IL_1B com_inf 7.266 2 60 1.493638e-03 dpi_within_group 4.117523e-03*

10 IL_1B com_uninf 1.411 2 60 2.518187e-01 dpi_within_group 3.299112e-01

11 IL_1B sup_inf 0.927 2 60 4.012251e-01 dpi_within_group 4.012251e-01

*12 IL_1B sup_uninf 7.998 2 60 8.331252e-04 dpi_within_group 4.117523e-03*

*13 IL_10 com_inf 6.713 2 60 2.338671e-03 dpi_within_group 3.274139e-03*

14 IL_10 com_uninf 0.440 2 60 6.462664e-01 dpi_within_group 6.462664e-01

*15 IL_10 sup_inf 10.570 2 60 1.167990e-04 dpi_within_group 2.725311e-04*

*16 IL_10 sup_uninf 7.605 2 60 1.138638e-03 dpi_within_group 1.992617e-03*

17 IL_17A com_inf 0.335 2 60 7.167350e-01 dpi_within_group 8.361908e-01

18 IL_17A com_uninf 0.790 2 60 4.586558e-01 dpi_within_group 6.577295e-01

19 IL_17A sup_inf 0.765 2 60 4.698068e-01 dpi_within_group 6.577295e-01

*20 IL_17A sup_uninf 6.988 2 60 1.869175e-03 dpi_within_group 6.542114e-03*

21 IL_18 com_inf 1.116 2 60 3.341475e-01 dpi_within_group 4.745287e-01

22 IL_18 com_uninf 1.795 2 60 1.749278e-01 dpi_within_group 4.081650e-01

*23 IL_18 sup_inf 25.093 2 60 1.204477e-08 dpi_within_group 5.382909e-08*

24 IL_18 sup_uninf 0.786 2 60 4.600862e-01 dpi_within_group 4.745287e-01

*25 IL_21 com_inf 3.601 2 60 3.335811e-02 dpi_within_group 7.783560e-02*

26 IL_21 com_uninf 0.012 2 60 9.885031e-01 dpi_within_group 9.885031e-01

27 IL_21 sup_inf 0.575 2 60 5.655262e-01 dpi_within_group 7.917367e-01

28 IL_21 sup_uninf 0.270 2 60 7.646095e-01 dpi_within_group 8.920444e-01

29 IL_23 com_inf 2.576 2 60 8.450007e-02 dpi_within_group 1.183001e-01

*30 IL_23 com_uninf 4.449 2 60 1.579190e-02 dpi_within_group 3.684776e-02*

*31 IL_23 sup_inf 15.023 2 60 5.136651e-06 dpi_within_group 1.797828e-05*

*32 IL_23 sup_uninf 4.021 2 60 2.297798e-02 dpi_within_group 4.021147e-02*

*33 INFgamma com_inf 8.084 2 60 7.785748e-04 dpi_within_group 2.725012e-03*

*34 INFgamma com_uninf 5.380 2 60 7.091957e-03 dpi_within_group 1.241092e-02*

*35 INFgamma sup_inf 5.488 2 60 6.473138e-03 dpi_within_group 1.241092e-02*

*36 INFgamma sup_uninf 3.578 2 60 3.404084e-02 dpi_within_group 4.765718e-02*

*37 TNFa com_inf 3.912 2 60 2.530425e-02 dpi_within_group 4.428244e-02*

38 TNFa com_uninf 2.222 2 60 1.172190e-01 dpi_within_group 1.445416e-01

39 TNFa sup_inf 0.531 2 60 5.908857e-01 dpi_within_group 5.908857e-01

40 TNFa sup_uninf 2.163 2 60 1.238928e-01 dpi_within_group 1.445416e-01

*41 MIP_2 com_inf 6.135 2 60 3.764952e-03 dpi_within_group 6.018855e-03*

*42 MIP_2 com_uninf 5.976 2 60 4.299182e-03 dpi_within_group 6.018855e-03*

*43 MIP_2 sup_inf 31.149 2 60 5.270582e-10 dpi_within_group 3.689408e-09*

*44 MIP_2 sup_uninf 5.135 2 60 8.735454e-03 dpi_within_group 1.019136e-02*

45 NLRP3 8 1.166 3 60 3.302606e-01 group_within_dpi 3.853041e-01

*46 NLRP3 16 3.835 3 60 1.402300e-02 group_within_dpi 1.963220e-02*

47 NLRP3 24 12.710 3 60 1.557136e-06 group_within_dpi 1.089995e-05

*48 PTGS2 8 12.854 3 60 1.369431e-06 group_within_dpi 4.793007e-06*

*49 PTGS2 16 15.293 3 60 1.674283e-07 group_within_dpi 1.171998e-06*

*50 PTGS2 24 5.516 3 60 2.059079e-03 group_within_dpi 3.603387e-03*

*51 IL_1B 8 5.397 3 60 2.352870e-03 group_within_dpi 4.117523e-03*

*52 IL_1B 16 5.600 3 60 1.875671e-03 group_within_dpi 4.117523e-03*

53 IL_1B 24 1.300 3 60 2.827811e-01 group_within_dpi 3.299112e-01

*54 IL_10 8 4.166 3 60 9.547388e-03 group_within_dpi 1.113862e-02*

*55 IL_10 16 9.207 3 60 4.231555e-05 group_within_dpi 2.725311e-04*

*56 IL_10 24 8.433 3 60 9.218365e-05 group_within_dpi 2.725311e-04*

57 IL_17A 8 0.265 3 60 8.500980e-01 group_within_dpi 8.500980e-01

58 IL_17A 16 1.831 3 60 1.510812e-01 group_within_dpi 3.525228e-01

*59 IL_17A 24 6.114 3 60 1.064680e-03 group_within_dpi 6.542114e-03*

60 IL_18 8 0.882 3 60 4.557878e-01 group_within_dpi 4.745287e-01

61 IL_18 16 0.845 3 60 4.745287e-01 group_within_dpi 4.745287e-01

*62 IL_18 24 18.274 3 60 1.537974e-08 group_within_dpi 5.382909e-08*

*63 IL_21 8 6.122 3 60 1.055592e-03 group_within_dpi 5.358488e-03*

*64 IL_21 16 5.783 3 60 1.530997e-03 group_within_dpi 5.358488e-03*

65 IL_21 24 1.405 3 60 2.501723e-01 group_within_dpi 4.378016e-01

*66 IL_23 8 23.890 3 60 2.691880e-10 group_within_dpi 1.884316e-09*

67 IL_23 16 1.392 3 60 2.539927e-01 group_within_dpi 2.963249e-01

68 IL_23 24 0.626 3 60 6.012184e-01 group_within_dpi 6.012184e-01

*69 INFgamma 8 11.240 3 60 5.970470e-06 group_within_dpi 4.179329e-05*

70 INFgamma 16 2.038 3 60 1.180498e-01 group_within_dpi 1.377248e-01

71 INFgamma 24 0.317 3 60 8.128533e-01 group_within_dpi 8.128533e-01

*72 TNFa 8 4.100 3 60 1.030405e-02 group_within_dpi 2.404278e-02*

*73 TNFa 16 7.564 3 60 2.258395e-04 group_within_dpi 1.580876e-03*

*74 TNFa 24 5.440 3 60 2.242272e-03 group_within_dpi 7.847953e-03*

*75 MIP_2 8 21.369 3 60 1.549085e-09 group_within_dpi 5.421797e-09*

*76 MIP_2 16 3.458 3 60 2.183038e-02 group_within_dpi 2.183038e-02*

*77 MIP_2 24 6.911 3 60 4.500292e-04 group_within_dpi 1.050068e-03*

With dpi_within_group contrasts, genes had two or more significant contrasts apart from four genes with only one significant contrast: IL-17A (CS group), IL-18 (AS group), IL-21 (A group), TNFa (A group). With group_within_dpi, genes had two or three significant contrasts apart from five genes with only one: NLRP3 (dpi 16), IL-17A (dpi 24), IL-18 (dpi 24), IL_23 (dpi 8), INFgamma (dpi 8).

Within the full contrast table (Table S4), for dpi_within_group the following genes had no statistically significant contrasts: IL-21, TNFa; the following gene had only one statistically significant contrast: PTGS2 (A group, dpi8 - dpi16); the following genes had only two statistically significant contrasts: IL_17A (CS group, dpi8 - dpi24 and CS group dpi16 - dpi24); and IL-18 (AS group, dpi8 - dpi24 and AS group, dpi16 - dpi24). For group_within_dpi all genes had three or more statistically significant contrasts.

**Table S4.** Results of generalized least squares regression (model: gls("log-fold data" ~ dpi * group with weights according to interaction between dpi and group). All contrasts are shown. Statistically significant contrasts are shown in red italics.

gene contrast_type group dpi contrast estimate SE df t.ratio p.value p_adj

1 NLRP3 dpi_within_group com_inf <NA> dpi8 - dpi16 0.815484690 0.5068583 60 1.608900802 1.128877e-01 1.935217e-01

*2 NLRP3 dpi_within_group com_inf <NA> dpi8 - dpi24 1.831729925 0.5068583 60 3.613889731 6.183494e-04 2.473398e-03*

3 NLRP3 dpi_within_group com_inf <NA> dpi16 - dpi24 1.016245235 0.5068583 60 2.004988929 4.948199e-02 9.896397e-02

4 NLRP3 dpi_within_group com_uninf <NA> dpi8 - dpi16 -0.521809861 0.5068583 60 -1.029498548 3.073778e-01 4.098371e-01

5 NLRP3 dpi_within_group com_uninf <NA> dpi8 - dpi24 -0.208451240 0.5068583 60 -0.411261390 6.823462e-01 6.823462e-01

6 NLRP3 dpi_within_group com_uninf <NA> dpi16 - dpi24 0.313358622 0.5068583 60 0.618237159 5.387589e-01 6.465107e-01

7 NLRP3 dpi_within_group sup_inf <NA> dpi8 - dpi16 0.218599014 0.5068583 60 0.431282320 6.678088e-01 6.823462e-01

*8 NLRP3 dpi_within_group sup_inf <NA> dpi8 - dpi24 1.625735488 0.5068583 60 3.207475461 2.149181e-03 6.447544e-03*

*9 NLRP3 dpi_within_group sup_inf <NA> dpi16 - dpi24 1.407136475 0.5068583 60 2.776193142 7.325550e-03 1.758132e-02*

10 NLRP3 dpi_within_group sup_uninf <NA> dpi8 - dpi16 0.549675528 0.5068583 60 1.084475784 2.824924e-01 4.098371e-01

*11 NLRP3 dpi_within_group sup_uninf <NA> dpi8 - dpi24 2.528603314 0.5068583 60 4.988777781 5.509495e-06 6.611394e-05*

*12 NLRP3 dpi_within_group sup_uninf <NA> dpi16 - dpi24 1.978927786 0.5068583 60 3.904301997 2.420761e-04 1.452457e-03*

*13 PTGS2 dpi_within_group com_inf <NA> dpi8 - dpi16 -1.405418887 0.3778337 60 -3.719675670 4.413223e-04 5.295868e-03*

14 PTGS2 dpi_within_group com_inf <NA> dpi8 - dpi24 -0.821763207 0.3778337 60 -2.174933491 3.358749e-02 8.064655e-02

15 PTGS2 dpi_within_group com_inf <NA> dpi16 - dpi24 0.583655681 0.3778337 60 1.544742179 1.276671e-01 2.188579e-01

16 PTGS2 dpi_within_group com_uninf <NA> dpi8 - dpi16 -0.821689874 0.3778337 60 -2.174739404 3.360273e-02 8.064655e-02

17 PTGS2 dpi_within_group com_uninf <NA> dpi8 - dpi24 -0.932994793 0.3778337 60 -2.469326451 1.640022e-02 8.064655e-02

18 PTGS2 dpi_within_group com_uninf <NA> dpi16 - dpi24 -0.111304919 0.3778337 60 -0.294587048 7.693262e-01 8.325553e-01

19 PTGS2 dpi_within_group sup_inf <NA> dpi8 - dpi16 0.321977391 0.3778337 60 0.852166907 3.975102e-01 5.300136e-01

20 PTGS2 dpi_within_group sup_inf <NA> dpi8 - dpi24 0.433798635 0.3778337 60 1.148120494 2.554758e-01 3.832138e-01

21 PTGS2 dpi_within_group sup_inf <NA> dpi16 - dpi24 0.111821244 0.3778337 60 0.295953587 7.682874e-01 8.325553e-01

22 PTGS2 dpi_within_group sup_uninf <NA> dpi8 - dpi16 0.824073086 0.3778337 60 2.181046971 3.311049e-02 8.064655e-02

23 PTGS2 dpi_within_group sup_uninf <NA> dpi8 - dpi24 0.080232243 0.3778337 60 0.212348024 8.325553e-01 8.325553e-01

24 PTGS2 dpi_within_group sup_uninf <NA> dpi16 - dpi24 -0.743840842 0.3778337 60 -1.968698947 5.361215e-02 1.072243e-01

*25 IL_1B dpi_within_group com_inf <NA> dpi8 - dpi16 -1.530299508 0.4164770 60 -3.674391254 5.101883e-04 3.061130e-03*

*26 IL_1B dpi_within_group com_inf <NA> dpi8 - dpi24 -1.131359370 0.4164770 60 -2.716498929 8.607707e-03 2.582312e-02*

27 IL_1B dpi_within_group com_inf <NA> dpi16 - dpi24 0.398940138 0.4164770 60 0.957892325 3.419611e-01 4.559481e-01

28 IL_1B dpi_within_group com_uninf <NA> dpi8 - dpi16 -0.519838819 0.4164770 60 -1.248181286 2.168136e-01 3.716805e-01

29 IL_1B dpi_within_group com_uninf <NA> dpi8 - dpi24 -0.665505349 0.4164770 60 -1.597940154 1.153099e-01 2.767437e-01

30 IL_1B dpi_within_group com_uninf <NA> dpi16 - dpi24 -0.145666530 0.4164770 60 -0.349758868 7.277444e-01 7.715182e-01

31 IL_1B dpi_within_group sup_inf <NA> dpi8 - dpi16 0.419035308 0.4164770 60 1.006142695 3.183875e-01 4.559481e-01

32 IL_1B dpi_within_group sup_inf <NA> dpi8 - dpi24 0.540523847 0.4164770 60 1.297847961 1.993069e-01 3.716805e-01

33 IL_1B dpi_within_group sup_inf <NA> dpi16 - dpi24 0.121488539 0.4164770 60 0.291705266 7.715182e-01 7.715182e-01

34 IL_1B dpi_within_group sup_uninf <NA> dpi8 - dpi16 -0.337331628 0.4164770 60 -0.809964570 4.211613e-01 5.053936e-01

*35 IL_1B dpi_within_group sup_uninf <NA> dpi8 - dpi24 -1.581341386 0.4164770 60 -3.796947542 3.438601e-04 3.061130e-03*

*36 IL_1B dpi_within_group sup_uninf <NA> dpi16 - dpi24 -1.244009758 0.4164770 60 -2.986982972 4.075904e-03 1.630362e-02*

*37 IL_10 dpi_within_group com_inf <NA> dpi8 - dpi16 1.573883055 0.5647047 60 2.787090338 7.111372e-03 1.422274e-02*

*38 IL_10 dpi_within_group com_inf <NA> dpi8 - dpi24 1.950274505 0.5647047 60 3.453618241 1.020542e-03 4.082168e-03*

39 IL_10 dpi_within_group com_inf <NA> dpi16 - dpi24 0.376391449 0.5647047 60 0.666527903 5.076287e-01 5.817393e-01

40 IL_10 dpi_within_group com_uninf <NA> dpi8 - dpi16 -0.518142305 0.5647047 60 -0.917545562 3.625305e-01 4.833740e-01

41 IL_10 dpi_within_group com_uninf <NA> dpi8 - dpi24 -0.353876377 0.5647047 60 -0.626657380 5.332610e-01 5.817393e-01

42 IL_10 dpi_within_group com_uninf <NA> dpi16 - dpi24 0.164265928 0.5647047 60 0.290888182 7.721400e-01 7.721400e-01

43 IL_10 dpi_within_group sup_inf <NA> dpi8 - dpi16 0.775859748 0.5647047 60 1.373921145 1.745774e-01 2.992756e-01

*44 IL_10 dpi_within_group sup_inf <NA> dpi8 - dpi24 2.533769182 0.5647047 60 4.486892202 3.330149e-05 3.996179e-04*

*45 IL_10 dpi_within_group sup_inf <NA> dpi16 - dpi24 1.757909434 0.5647047 60 3.112971057 2.836706e-03 8.510117e-03*

*46 IL_10 dpi_within_group sup_uninf <NA> dpi8 - dpi16 1.578826740 0.5647047 60 2.795844798 6.943499e-03 1.422274e-02*

*47 IL_10 dpi_within_group sup_uninf <NA> dpi8 - dpi24 2.119102590 0.5647047 60 3.752585260 3.969538e-04 2.381723e-03*

48 IL_10 dpi_within_group sup_uninf <NA> dpi16 - dpi24 0.540275850 0.5647047 60 0.956740461 3.425375e-01 4.833740e-01

49 IL_17A dpi_within_group com_inf <NA> dpi8 - dpi16 0.266135321 0.6221404 60 0.427773764 6.703473e-01 7.521921e-01

50 IL_17A dpi_within_group com_inf <NA> dpi8 - dpi24 0.508993997 0.6221404 60 0.818133712 4.165182e-01 6.378688e-01

51 IL_17A dpi_within_group com_inf <NA> dpi16 - dpi24 0.242858676 0.6221404 60 0.390359948 6.976529e-01 7.521921e-01

52 IL_17A dpi_within_group com_uninf <NA> dpi8 - dpi16 -0.764330278 0.6221404 60 -1.228549592 2.240378e-01 6.378688e-01

53 IL_17A dpi_within_group com_uninf <NA> dpi8 - dpi24 -0.239610088 0.6221404 60 -0.385138315 7.014969e-01 7.521921e-01

54 IL_17A dpi_within_group com_uninf <NA> dpi16 - dpi24 0.524720190 0.6221404 60 0.843411277 4.023484e-01 6.378688e-01

55 IL_17A dpi_within_group sup_inf <NA> dpi8 - dpi16 0.197343671 0.6221404 60 0.317201206 7.521921e-01 7.521921e-01

56 IL_17A dpi_within_group sup_inf <NA> dpi8 - dpi24 0.742842762 0.6221404 60 1.194011539 2.371728e-01 6.378688e-01

57 IL_17A dpi_within_group sup_inf <NA> dpi16 - dpi24 0.545499091 0.6221404 60 0.876810333 3.840869e-01 6.378688e-01

58 IL_17A dpi_within_group sup_uninf <NA> dpi8 - dpi16 0.499468566 0.6221404 60 0.802822968 4.252459e-01 6.378688e-01

*59 IL_17A dpi_within_group sup_uninf <NA> dpi8 - dpi24 2.217045656 0.6221404 60 3.563577962 7.246233e-04 8.695479e-03*

*60 IL_17A dpi_within_group sup_uninf <NA> dpi16 - dpi24 1.717577090 0.6221404 60 2.760754994 7.639143e-03 4.583486e-02*

61 IL_18 dpi_within_group com_inf <NA> dpi8 - dpi16 -0.268016910 0.3821503 60 -0.701339053 4.858028e-01 5.829634e-01

62 IL_18 dpi_within_group com_inf <NA> dpi8 - dpi24 -0.570691179 0.3821503 60 -1.493368501 1.405811e-01 4.217433e-01

63 IL_18 dpi_within_group com_inf <NA> dpi16 - dpi24 -0.302674269 0.3821503 60 -0.792029448 4.314638e-01 5.752851e-01

64 IL_18 dpi_within_group com_uninf <NA> dpi8 - dpi16 -0.721847105 0.3821503 60 -1.888909044 6.373999e-02 2.549599e-01

65 IL_18 dpi_within_group com_uninf <NA> dpi8 - dpi24 -0.311735023 0.3821503 60 -0.815739373 4.178759e-01 5.752851e-01

66 IL_18 dpi_within_group com_uninf <NA> dpi16 - dpi24 0.410112082 0.3821503 60 1.073169672 2.874923e-01 5.749846e-01

67 IL_18 dpi_within_group sup_inf <NA> dpi8 - dpi16 0.003568375 0.3821503 60 0.009337623 9.925807e-01 9.925807e-01

*68 IL_18 dpi_within_group sup_inf <NA> dpi8 - dpi24 2.346303206 0.3821503 60 6.139739727 7.169582e-08 4.459739e-07*

*69 IL_18 dpi_within_group sup_inf <NA> dpi16 - dpi24 2.342734831 0.3821503 60 6.130402104 7.432899e-08 4.459739e-07*

70 IL_18 dpi_within_group sup_uninf <NA> dpi8 - dpi16 0.092871270 0.3821503 60 0.243022906 8.088166e-01 8.823453e-01

71 IL_18 dpi_within_group sup_uninf <NA> dpi8 - dpi24 0.453639180 0.3821503 60 1.187070148 2.398786e-01 5.749846e-01

72 IL_18 dpi_within_group sup_uninf <NA> dpi16 - dpi24 0.360767910 0.3821503 60 0.944047242 3.489316e-01 5.752851e-01

73 IL_21 dpi_within_group com_inf <NA> dpi8 - dpi16 0.406432511 0.5975799 60 0.680130817 4.990376e-01 9.609668e-01

74 IL_21 dpi_within_group com_inf <NA> dpi8 - dpi24 1.546650319 0.5975799 60 2.588189961 1.208574e-02 1.450289e-01

75 IL_21 dpi_within_group com_inf <NA> dpi16 - dpi24 1.140217808 0.5975799 60 1.908059144 6.117192e-02 3.670315e-01

76 IL_21 dpi_within_group com_uninf <NA> dpi8 - dpi16 0.059803186 0.5975799 60 0.100075631 9.206179e-01 9.609668e-01

77 IL_21 dpi_within_group com_uninf <NA> dpi8 - dpi24 0.089171386 0.5975799 60 0.149220856 8.818798e-01 9.609668e-01

78 IL_21 dpi_within_group com_uninf <NA> dpi16 - dpi24 0.029368199 0.5975799 60 0.049145225 9.609668e-01 9.609668e-01

79 IL_21 dpi_within_group sup_inf <NA> dpi8 - dpi16 -0.451439427 0.5975799 60 -0.755446126 4.529369e-01 9.609668e-01

80 IL_21 dpi_within_group sup_inf <NA> dpi8 - dpi24 0.168478335 0.5975799 60 0.281934403 7.789640e-01 9.609668e-01

81 IL_21 dpi_within_group sup_inf <NA> dpi16 - dpi24 0.619917761 0.5975799 60 1.037380529 3.037213e-01 9.609668e-01

82 IL_21 dpi_within_group sup_uninf <NA> dpi8 - dpi16 -0.067672890 0.5975799 60 -0.113244923 9.102144e-01 9.609668e-01

83 IL_21 dpi_within_group sup_uninf <NA> dpi8 - dpi24 -0.409301646 0.5975799 60 -0.684932073 4.960243e-01 9.609668e-01

84 IL_21 dpi_within_group sup_uninf <NA> dpi16 - dpi24 -0.341628755 0.5975799 60 -0.571687151 5.696695e-01 9.609668e-01

85 IL_23 dpi_within_group com_inf <NA> dpi8 - dpi16 -1.304719279 0.5821755 60 -2.241109809 2.873135e-02 5.746270e-02

86 IL_23 dpi_within_group com_inf <NA> dpi8 - dpi24 -0.833208986 0.5821755 60 -1.431198926 1.575614e-01 2.213046e-01

87 IL_23 dpi_within_group com_inf <NA> dpi16 - dpi24 0.471510293 0.5821755 60 0.809910883 4.211920e-01 4.594821e-01

*88 IL_23 dpi_within_group com_uninf <NA> dpi8 - dpi16 -1.392443169 0.5821755 60 -2.391792700 1.991717e-02 4.780120e-02*

89 IL_23 dpi_within_group com_uninf <NA> dpi8 - dpi24 0.202408902 0.5821755 60 0.347676764 7.292997e-01 7.292997e-01

*90 IL_23 dpi_within_group com_uninf <NA> dpi16 - dpi24 1.594852072 0.5821755 60 2.739469463 8.091757e-03 2.427527e-02*

*91 IL_23 dpi_within_group sup_inf <NA> dpi8 - dpi16 2.268199371 0.5821755 60 3.896074768 2.487188e-04 1.492313e-03*

*92 IL_23 dpi_within_group sup_inf <NA> dpi8 - dpi24 3.078039523 0.5821755 60 5.287133164 1.831967e-06 2.198361e-05*

93 IL_23 dpi_within_group sup_inf <NA> dpi16 - dpi24 0.809840152 0.5821755 60 1.391058397 1.693449e-01 2.213046e-01

94 IL_23 dpi_within_group sup_uninf <NA> dpi8 - dpi16 0.781698757 0.5821755 60 1.342720063 1.844205e-01 2.213046e-01

*95 IL_23 dpi_within_group sup_uninf <NA> dpi8 - dpi24 1.650151444 0.5821755 60 2.834456919 6.245745e-03 2.427527e-02*

96 IL_23 dpi_within_group sup_uninf <NA> dpi16 - dpi24 0.868452687 0.5821755 60 1.491736856 1.410075e-01 2.213046e-01

97 INFgamma dpi_within_group com_inf <NA> dpi8 - dpi16 1.342796178 0.6580461 60 2.040580784 4.570005e-02 7.834294e-02

*98 INFgamma dpi_within_group com_inf <NA> dpi8 - dpi24 2.645896917 0.6580461 60 4.020838378 1.644829e-04 1.973795e-03*

99 INFgamma dpi_within_group com_inf <NA> dpi16 - dpi24 1.303100739 0.6580461 60 1.980257594 5.226573e-02 7.839860e-02

*100 INFgamma dpi_within_group com_uninf <NA> dpi8 - dpi16 -1.989530952 0.6580461 60 -3.023391560 3.673905e-03 1.469562e-02*

101 INFgamma dpi_within_group com_uninf <NA> dpi8 - dpi24 -0.269455264 0.6580461 60 -0.409477806 6.836472e-01 6.836472e-01

*102 INFgamma dpi_within_group com_uninf <NA> dpi16 - dpi24 1.720075689 0.6580461 60 2.613913753 1.129996e-02 2.711990e-02*

103 INFgamma dpi_within_group sup_inf <NA> dpi8 - dpi16 -0.775641449 0.6580461 60 -1.178703858 2.431695e-01 2.652758e-01

104 INFgamma dpi_within_group sup_inf <NA> dpi8 - dpi24 1.376713319 0.6580461 60 2.092122982 4.066692e-02 7.834294e-02

*105 INFgamma dpi_within_group sup_inf <NA> dpi16 - dpi24 2.152354768 0.6580461 60 3.270826840 1.779572e-03 1.067743e-02*

*106 INFgamma dpi_within_group sup_uninf <NA> dpi8 - dpi16 -1.759445611 0.6580461 60 -2.673742272 9.649097e-03 2.711990e-02*

107 INFgamma dpi_within_group sup_uninf <NA> dpi8 - dpi24 -0.831824524 0.6580461 60 -1.264082492 2.110893e-01 2.533071e-01

108 INFgamma dpi_within_group sup_uninf <NA> dpi16 - dpi24 0.927621087 0.6580461 60 1.409659780 1.638027e-01 2.184036e-01

109 TNFa dpi_within_group com_inf <NA> dpi8 - dpi16 1.463618621 0.6057054 60 2.416386916 1.873459e-02 1.124075e-01

110 TNFa dpi_within_group com_inf <NA> dpi8 - dpi24 1.470717515 0.6057054 60 2.428106959 1.819351e-02 1.124075e-01

111 TNFa dpi_within_group com_inf <NA> dpi16 - dpi24 0.007098894 0.6057054 60 0.011720043 9.906879e-01 9.906879e-01

112 TNFa dpi_within_group com_uninf <NA> dpi8 - dpi16 -1.181631865 0.6057054 60 -1.950835920 5.575129e-02 1.672539e-01

113 TNFa dpi_within_group com_uninf <NA> dpi8 - dpi24 -0.171643538 0.6057054 60 -0.283377919 7.778626e-01 9.220062e-01

114 TNFa dpi_within_group com_uninf <NA> dpi16 - dpi24 1.009988328 0.6057054 60 1.667458001 1.006352e-01 2.415244e-01

115 TNFa dpi_within_group sup_inf <NA> dpi8 - dpi16 -0.118796096 0.6057054 60 -0.196128505 8.451724e-01 9.220062e-01

116 TNFa dpi_within_group sup_inf <NA> dpi8 - dpi24 0.471177118 0.6057054 60 0.777898154 4.396858e-01 6.583887e-01

117 TNFa dpi_within_group sup_inf <NA> dpi16 - dpi24 0.589973214 0.6057054 60 0.974026658 3.339540e-01 5.724925e-01

118 TNFa dpi_within_group sup_uninf <NA> dpi8 - dpi16 0.820933462 0.6057054 60 1.355334544 1.803914e-01 3.607828e-01

119 TNFa dpi_within_group sup_uninf <NA> dpi8 - dpi24 1.237961662 0.6057054 60 2.043834589 4.536710e-02 1.672539e-01

120 TNFa dpi_within_group sup_uninf <NA> dpi16 - dpi24 0.417028200 0.6057054 60 0.688500044 4.937915e-01 6.583887e-01

*121 MIP_2 dpi_within_group com_inf <NA> dpi8 - dpi16 -1.795237382 0.5858434 60 -3.064364018 3.265844e-03 6.531687e-03*

122 MIP_2 dpi_within_group com_inf <NA> dpi8 - dpi24 -0.036626958 0.5858434 60 -0.062520051 9.503565e-01 9.503565e-01

*123 MIP_2 dpi_within_group com_inf <NA> dpi16 - dpi24 1.758610423 0.5858434 60 3.001843967 3.907113e-03 6.697908e-03*

*124 MIP_2 dpi_within_group com_uninf <NA> dpi8 - dpi16 -1.873832957 0.5858434 60 -3.198522016 2.206897e-03 6.530336e-03*

*125 MIP_2 dpi_within_group com_uninf <NA> dpi8 - dpi24 -1.602444855 0.5858434 60 -2.735278580 8.183720e-03 1.227558e-02*

126 MIP_2 dpi_within_group com_uninf <NA> dpi16 - dpi24 0.271388102 0.5858434 60 0.463243436 6.448659e-01 7.034900e-01

*127 MIP_2 dpi_within_group sup_inf <NA> dpi8 - dpi16 2.328242935 0.5858434 60 3.974172969 1.921389e-04 9.233503e-04*

*128 MIP_2 dpi_within_group sup_inf <NA> dpi8 - dpi24 4.624004248 0.5858434 60 7.892901731 7.464228e-11 8.957074e-10*

*129 MIP_2 dpi_within_group sup_inf <NA> dpi16 - dpi24 2.295761313 0.5858434 60 3.918728762 2.308376e-04 9.233503e-04*

130 MIP_2 dpi_within_group sup_uninf <NA> dpi8 - dpi16 0.560501671 0.5858434 60 0.956743198 3.425361e-01 4.110433e-01

*131 MIP_2 dpi_within_group sup_uninf <NA> dpi8 - dpi24 1.832083770 0.5858434 60 3.127258623 2.720973e-03 6.530336e-03*

*132 MIP_2 dpi_within_group sup_uninf <NA> dpi16 - dpi24 1.271582099 0.5858434 60 2.170515425 3.393595e-02 4.524793e-02*

133 NLRP3 group_within_dpi <NA> 8 com_inf - com_uninf 0.202743712 0.5068583 60 0.400000791 6.905765e-01 7.311986e-01

134 NLRP3 group_within_dpi <NA> 8 com_inf - sup_inf -0.386441376 0.5068583 60 -0.762424908 4.487934e-01 5.770201e-01

135 NLRP3 group_within_dpi <NA> 8 com_inf - sup_uninf 0.538896303 0.5068583 60 1.063209039 2.919477e-01 4.042352e-01

136 NLRP3 group_within_dpi <NA> 8 com_uninf - sup_inf -0.589185087 0.5068583 60 -1.162425698 2.496654e-01 3.744981e-01

137 NLRP3 group_within_dpi <NA> 8 com_uninf - sup_uninf 0.336152591 0.5068583 60 0.663208249 5.097373e-01 6.116847e-01

138 NLRP3 group_within_dpi <NA> 8 sup_inf - sup_uninf 0.925337678 0.5068583 60 1.825633947 7.288310e-02 1.311896e-01

139 NLRP3 group_within_dpi <NA> 16 com_inf - com_uninf -1.134550839 0.5068583 60 -2.238398560 2.891743e-02 6.506421e-02

140 NLRP3 group_within_dpi <NA> 16 com_inf - sup_inf -0.983327052 0.5068583 60 -1.940043390 5.707871e-02 1.141574e-01

141 NLRP3 group_within_dpi <NA> 16 com_inf - sup_uninf 0.273087141 0.5068583 60 0.538784021 5.920294e-01 6.660331e-01

142 NLRP3 group_within_dpi <NA> 16 com_uninf - sup_inf 0.151223787 0.5068583 60 0.298355170 7.664629e-01 7.664629e-01

*143 NLRP3 group_within_dpi <NA> 16 com_uninf - sup_uninf 1.407637980 0.5068583 60 2.777182581 7.305861e-03 3.287637e-02*

*144 NLRP3 group_within_dpi <NA> 16 sup_inf - sup_uninf 1.256414193 0.5068583 60 2.478827412 1.601014e-02 4.562594e-02*

*145 NLRP3 group_within_dpi <NA> 24 com_inf - com_uninf -1.837437453 0.5068583 60 -3.625150330 5.966885e-04 3.792585e-03*

146 NLRP3 group_within_dpi <NA> 24 com_inf - sup_inf -0.592435812 0.5068583 60 -1.168839177 2.470914e-01 3.744981e-01

*147 NLRP3 group_within_dpi <NA> 24 com_inf - sup_uninf 1.235769692 0.5068583 60 2.438097089 1.774342e-02 4.562594e-02*

*148 NLRP3 group_within_dpi <NA> 24 com_uninf - sup_inf 1.245001640 0.5068583 60 2.456311153 1.694848e-02 4.562594e-02*

*149 NLRP3 group_within_dpi <NA> 24 com_uninf - sup_uninf 3.073207144 0.5068583 60 6.063247419 9.631251e-08 1.733625e-06*

*150 NLRP3 group_within_dpi <NA> 24 sup_inf - sup_uninf 1.828205504 0.5068583 60 3.606936266 6.320975e-04 3.792585e-03*

*151 PTGS2 group_within_dpi <NA> 8 com_inf - com_uninf 1.642292086 0.3778337 60 4.346600128 5.430283e-05 1.954902e-04*

152 PTGS2 group_within_dpi <NA> 8 com_inf - sup_inf -0.556628815 0.3778337 60 -1.473211069 1.459212e-01 1.876130e-01

153 PTGS2 group_within_dpi <NA> 8 com_inf - sup_uninf -0.067633478 0.3778337 60 -0.179003289 8.585382e-01 8.657253e-01

*154 PTGS2 group_within_dpi <NA> 8 com_uninf - sup_inf -2.198920901 0.3778337 60 -5.819811197 2.451681e-07 2.138330e-06*

*155 PTGS2 group_within_dpi <NA> 8 com_uninf - sup_uninf -1.709925564 0.3778337 60 -4.525603417 2.906477e-05 1.307915e-04*

156 PTGS2 group_within_dpi <NA> 8 sup_inf - sup_uninf 0.488995337 0.3778337 60 1.294207780 2.005530e-01 2.406635e-01

*157 PTGS2 group_within_dpi <NA> 16 com_inf - com_uninf 2.226021099 0.3778337 60 5.891536394 1.863265e-07 2.138330e-06*

*158 PTGS2 group_within_dpi <NA> 16 com_inf - sup_inf 1.170767463 0.3778337 60 3.098631509 2.957479e-03 7.604946e-03*

*159 PTGS2 group_within_dpi <NA> 16 com_inf - sup_uninf 2.161858495 0.3778337 60 5.721719352 3.563883e-07 2.138330e-06*

*160 PTGS2 group_within_dpi <NA> 16 com_uninf - sup_inf -1.055253636 0.3778337 60 -2.792904886 6.999463e-03 1.574879e-02*

161 PTGS2 group_within_dpi <NA> 16 com_uninf - sup_uninf -0.064162604 0.3778337 60 -0.169817042 8.657253e-01 8.657253e-01

*162 PTGS2 group_within_dpi <NA> 16 sup_inf - sup_uninf 0.991091032 0.3778337 60 2.623087844 1.103116e-02 2.206232e-02*

*163 PTGS2 group_within_dpi <NA> 24 com_inf - com_uninf 1.531060499 0.3778337 60 4.052207167 1.480942e-04 4.442825e-04*

164 PTGS2 group_within_dpi <NA> 24 com_inf - sup_inf 0.698933026 0.3778337 60 1.849842916 6.926260e-02 9.710692e-02

165 PTGS2 group_within_dpi <NA> 24 com_inf - sup_uninf 0.834361972 0.3778337 60 2.208278226 3.105725e-02 5.153464e-02

166 PTGS2 group_within_dpi <NA> 24 com_uninf - sup_inf -0.832127473 0.3778337 60 -2.202364251 3.149339e-02 5.153464e-02

167 PTGS2 group_within_dpi <NA> 24 com_uninf - sup_uninf -0.696698527 0.3778337 60 -1.843928942 7.013277e-02 9.710692e-02

168 PTGS2 group_within_dpi <NA> 24 sup_inf - sup_uninf 0.135428946 0.3778337 60 0.358435310 7.212757e-01 8.114351e-01

169 IL_1B group_within_dpi <NA> 8 com_inf - com_uninf 0.147213866 0.4164770 60 0.353474165 7.249720e-01 8.155935e-01

*170 IL_1B group_within_dpi <NA> 8 com_inf - sup_inf -1.106509612 0.4164770 60 -2.656832351 1.009182e-02 3.633056e-02*

171 IL_1B group_within_dpi <NA> 8 com_inf - sup_uninf 0.467500126 0.4164770 60 1.122511224 2.661170e-01 3.684697e-01

*172 IL_1B group_within_dpi <NA> 8 com_uninf - sup_inf -1.253723478 0.4164770 60 -3.010306516 3.813926e-03 2.288356e-02*

173 IL_1B group_within_dpi <NA> 8 com_uninf - sup_uninf 0.320286260 0.4164770 60 0.769037060 4.448881e-01 5.431411e-01

*174 IL_1B group_within_dpi <NA> 8 sup_inf - sup_uninf 1.574009738 0.4164770 60 3.779343576 3.640590e-04 3.276531e-03*

*175 IL_1B group_within_dpi <NA> 16 com_inf - com_uninf 1.157674555 0.4164770 60 2.779684133 7.256299e-03 3.265335e-02*

176 IL_1B group_within_dpi <NA> 16 com_inf - sup_inf 0.842825205 0.4164770 60 2.023701598 4.746132e-02 1.395224e-01

*177 IL_1B group_within_dpi <NA> 16 com_inf - sup_uninf 1.660468007 0.4164770 60 3.986937908 1.841580e-04 3.276531e-03*

178 IL_1B group_within_dpi <NA> 16 com_uninf - sup_inf -0.314849351 0.4164770 60 -0.755982535 4.526176e-01 5.431411e-01

179 IL_1B group_within_dpi <NA> 16 com_uninf - sup_uninf 0.502793451 0.4164770 60 1.207253776 2.320722e-01 3.481083e-01

180 IL_1B group_within_dpi <NA> 16 sup_inf - sup_uninf 0.817642802 0.4164770 60 1.963236311 5.425873e-02 1.395224e-01

181 IL_1B group_within_dpi <NA> 24 com_inf - com_uninf 0.613067887 0.4164770 60 1.472032940 1.462382e-01 3.158248e-01

182 IL_1B group_within_dpi <NA> 24 com_inf - sup_inf 0.565373605 0.4164770 60 1.357514538 1.797019e-01 3.164119e-01

183 IL_1B group_within_dpi <NA> 24 com_inf - sup_uninf 0.017518111 0.4164770 60 0.042062611 9.665884e-01 9.665884e-01

184 IL_1B group_within_dpi <NA> 24 com_uninf - sup_inf -0.047694282 0.4164770 60 -0.114518401 9.092092e-01 9.626921e-01

185 IL_1B group_within_dpi <NA> 24 com_uninf - sup_uninf -0.595549777 0.4164770 60 -1.429970329 1.579124e-01 3.158248e-01

186 IL_1B group_within_dpi <NA> 24 sup_inf - sup_uninf -0.547855495 0.4164770 60 -1.315451928 1.933628e-01 3.164119e-01

187 IL_10 group_within_dpi <NA> 8 com_inf - com_uninf 0.881622729 0.5647047 60 1.561210143 1.237337e-01 1.590862e-01

188 IL_10 group_within_dpi <NA> 8 com_inf - sup_inf -0.538650239 0.5647047 60 -0.953861770 3.439808e-01 4.127770e-01

*189 IL_10 group_within_dpi <NA> 8 com_inf - sup_uninf 1.248347590 0.5647047 60 2.210620093 3.088602e-02 4.632903e-02*

*190 IL_10 group_within_dpi <NA> 8 com_uninf - sup_inf -1.420272968 0.5647047 60 -2.515071913 1.459770e-02 3.330887e-02*

191 IL_10 group_within_dpi <NA> 8 com_uninf - sup_uninf 0.366724861 0.5647047 60 0.649409950 5.185518e-01 5.833708e-01

*192 IL_10 group_within_dpi <NA> 8 sup_inf - sup_uninf 1.786997829 0.5647047 60 3.164481864 2.439886e-03 1.097949e-02*

193 IL_10 group_within_dpi <NA> 16 com_inf - com_uninf -1.210402632 0.5647047 60 -2.143425758 3.614261e-02 5.004361e-02

*194 IL_10 group_within_dpi <NA> 16 com_inf - sup_inf -1.336673546 0.5647047 60 -2.367030964 2.117485e-02 3.811473e-02*

*195 IL_10 group_within_dpi <NA> 16 com_inf - sup_uninf 1.253291275 0.5647047 60 2.219374553 3.025325e-02 4.632903e-02*

196 IL_10 group_within_dpi <NA> 16 com_uninf - sup_inf -0.126270915 0.5647047 60 -0.223605207 8.238241e-01 8.722844e-01

*197 IL_10 group_within_dpi <NA> 16 com_uninf - sup_uninf 2.463693906 0.5647047 60 4.362800311 5.133919e-05 3.080351e-04*

*198 IL_10 group_within_dpi <NA> 16 sup_inf - sup_uninf 2.589964821 0.5647047 60 4.586405517 2.344905e-05 2.110414e-04*

*199 IL_10 group_within_dpi <NA> 24 com_inf - com_uninf -1.422528153 0.5647047 60 -2.519065478 1.444916e-02 3.330887e-02*

200 IL_10 group_within_dpi <NA> 24 com_inf - sup_inf 0.044844438 0.5647047 60 0.079412190 9.369690e-01 9.369690e-01

*201 IL_10 group_within_dpi <NA> 24 com_inf - sup_uninf 1.417175675 0.5647047 60 2.509587112 1.480394e-02 3.330887e-02*

*202 IL_10 group_within_dpi <NA> 24 com_uninf - sup_inf 1.467372591 0.5647047 60 2.598477668 1.176571e-02 3.330887e-02*

*203 IL_10 group_within_dpi <NA> 24 com_uninf - sup_uninf 2.839703828 0.5647047 60 5.028652590 4.761426e-06 8.570567e-05*

*204 IL_10 group_within_dpi <NA> 24 sup_inf - sup_uninf 1.372331237 0.5647047 60 2.430174922 1.809951e-02 3.619903e-02*

205 IL_17A group_within_dpi <NA> 8 com_inf - com_uninf -0.065898113 0.6221404 60 -0.105921618 9.159979e-01 9.698801e-01

206 IL_17A group_within_dpi <NA> 8 com_inf - sup_inf -0.467931703 0.6221404 60 -0.752132054 4.549122e-01 6.823683e-01

207 IL_17A group_within_dpi <NA> 8 com_inf - sup_uninf 0.007347279 0.6221404 60 0.011809681 9.906166e-01 9.906166e-01

208 IL_17A group_within_dpi <NA> 8 com_uninf - sup_inf -0.402033590 0.6221404 60 -0.646210436 5.206072e-01 7.208408e-01

209 IL_17A group_within_dpi <NA> 8 com_uninf - sup_uninf 0.073245392 0.6221404 60 0.117731298 9.066738e-01 9.698801e-01

210 IL_17A group_within_dpi <NA> 8 sup_inf - sup_uninf 0.475278981 0.6221404 60 0.763941735 4.478958e-01 6.823683e-01

211 IL_17A group_within_dpi <NA> 16 com_inf - com_uninf -1.096363712 0.6221404 60 -1.762244974 8.312130e-02 2.992367e-01

212 IL_17A group_within_dpi <NA> 16 com_inf - sup_inf -0.536723353 0.6221404 60 -0.862704612 3.917351e-01 6.823683e-01

213 IL_17A group_within_dpi <NA> 16 com_inf - sup_uninf 0.240680524 0.6221404 60 0.386858885 7.002294e-01 8.496661e-01

214 IL_17A group_within_dpi <NA> 16 com_uninf - sup_inf 0.559640359 0.6221404 60 0.899540362 3.719610e-01 6.823683e-01

215 IL_17A group_within_dpi <NA> 16 com_uninf - sup_uninf 1.337044236 0.6221404 60 2.149103858 3.566996e-02 1.605148e-01

216 IL_17A group_within_dpi <NA> 16 sup_inf - sup_uninf 0.777403876 0.6221404 60 1.249563497 2.163115e-01 5.562297e-01

217 IL_17A group_within_dpi <NA> 24 com_inf - com_uninf -0.814502198 0.6221404 60 -1.309193645 1.954605e-01 5.562297e-01

218 IL_17A group_within_dpi <NA> 24 com_inf - sup_inf -0.234082938 0.6221404 60 -0.376254227 7.080551e-01 8.496661e-01

*219 IL_17A group_within_dpi <NA> 24 com_inf - sup_uninf 1.715398937 0.6221404 60 2.757253931 7.711954e-03 4.627172e-02*

220 IL_17A group_within_dpi <NA> 24 com_uninf - sup_inf 0.580419260 0.6221404 60 0.932939418 3.545905e-01 6.823683e-01

*221 IL_17A group_within_dpi <NA> 24 com_uninf - sup_uninf 2.529901135 0.6221404 60 4.066447576 1.411852e-04 2.541333e-03*

*222 IL_17A group_within_dpi <NA> 24 sup_inf - sup_uninf 1.949481876 0.6221404 60 3.133508158 2.671754e-03 2.404578e-02*

223 IL_18 group_within_dpi <NA> 8 com_inf - com_uninf -0.130723927 0.3821503 60 -0.342074668 7.334900e-01 8.306745e-01

224 IL_18 group_within_dpi <NA> 8 com_inf - sup_inf -0.438993214 0.3821503 60 -1.148745001 2.552202e-01 5.257277e-01

225 IL_18 group_within_dpi <NA> 8 com_inf - sup_uninf -0.539241440 0.3821503 60 -1.411071717 1.633878e-01 5.257277e-01

226 IL_18 group_within_dpi <NA> 8 com_uninf - sup_inf -0.308269287 0.3821503 60 -0.806670333 4.230425e-01 6.345637e-01

227 IL_18 group_within_dpi <NA> 8 com_uninf - sup_uninf -0.408517513 0.3821503 60 -1.068997049 2.893529e-01 5.257277e-01

228 IL_18 group_within_dpi <NA> 8 sup_inf - sup_uninf -0.100248226 0.3821503 60 -0.262326716 7.939682e-01 8.406722e-01

229 IL_18 group_within_dpi <NA> 16 com_inf - com_uninf -0.584554122 0.3821503 60 -1.529644659 1.313602e-01 5.257277e-01

230 IL_18 group_within_dpi <NA> 16 com_inf - sup_inf -0.167407929 0.3821503 60 -0.438068324 6.629099e-01 8.306745e-01

231 IL_18 group_within_dpi <NA> 16 com_inf - sup_uninf -0.178353261 0.3821503 60 -0.466709758 6.423978e-01 8.306745e-01

232 IL_18 group_within_dpi <NA> 16 com_uninf - sup_inf 0.417146193 0.3821503 60 1.091576334 2.793833e-01 5.257277e-01

233 IL_18 group_within_dpi <NA> 16 com_uninf - sup_uninf 0.406200861 0.3821503 60 1.062934901 2.920709e-01 5.257277e-01

234 IL_18 group_within_dpi <NA> 16 sup_inf - sup_uninf -0.010945332 0.3821503 60 -0.028641433 9.772456e-01 9.772456e-01

235 IL_18 group_within_dpi <NA> 24 com_inf - com_uninf 0.128232228 0.3821503 60 0.335554461 7.383774e-01 8.306745e-01

*236 IL_18 group_within_dpi <NA> 24 com_inf - sup_inf 2.478001171 0.3821503 60 6.484363228 1.882040e-08 3.387673e-07*

237 IL_18 group_within_dpi <NA> 24 com_inf - sup_uninf 0.485088918 0.3821503 60 1.269366932 2.092119e-01 5.257277e-01

*238 IL_18 group_within_dpi <NA> 24 com_uninf - sup_inf 2.349768942 0.3821503 60 6.148808767 6.922706e-08 6.230435e-07*

239 IL_18 group_within_dpi <NA> 24 com_uninf - sup_uninf 0.356856690 0.3821503 60 0.933812472 3.541435e-01 5.795076e-01

*240 IL_18 group_within_dpi <NA> 24 sup_inf - sup_uninf -1.992912252 0.3821503 60 -5.214996295 2.395269e-06 1.437161e-05*

*241 IL_21 group_within_dpi <NA> 8 com_inf - com_uninf 1.795924563 0.5975799 60 3.005329561 3.868477e-03 1.740815e-02*

242 IL_21 group_within_dpi <NA> 8 com_inf - sup_inf 0.651205834 0.5975799 60 1.089738502 2.801858e-01 4.202786e-01

*243 IL_21 group_within_dpi <NA> 8 com_inf - sup_uninf 2.288053923 0.5975799 60 3.828866887 3.099444e-04 5.579000e-03*

244 IL_21 group_within_dpi <NA> 8 com_uninf - sup_inf -1.144718729 0.5975799 60 -1.915591059 6.018623e-02 1.354190e-01

245 IL_21 group_within_dpi <NA> 8 com_uninf - sup_uninf 0.492129360 0.5975799 60 0.823537327 4.134640e-01 5.724886e-01

*246 IL_21 group_within_dpi <NA> 8 sup_inf - sup_uninf 1.636848089 0.5975799 60 2.739128386 8.099206e-03 2.429762e-02*

*247 IL_21 group_within_dpi <NA> 16 com_inf - com_uninf 1.449295238 0.5975799 60 2.425274375 1.832298e-02 4.711624e-02*

248 IL_21 group_within_dpi <NA> 16 com_inf - sup_inf -0.206666104 0.5975799 60 -0.345838441 7.306738e-01 7.736547e-01

*249 IL_21 group_within_dpi <NA> 16 com_inf - sup_uninf 1.813948521 0.5975799 60 3.035491148 3.548706e-03 1.740815e-02*

*250 IL_21 group_within_dpi <NA> 16 com_uninf - sup_inf -1.655961342 0.5975799 60 -2.771112816 7.427415e-03 2.429762e-02*

251 IL_21 group_within_dpi <NA> 16 com_uninf - sup_uninf 0.364653283 0.5975799 60 0.610216773 5.440226e-01 6.530082e-01

*252 IL_21 group_within_dpi <NA> 16 sup_inf - sup_uninf 2.020614625 0.5975799 60 3.381329589 1.274102e-03 1.146692e-02*

253 IL_21 group_within_dpi <NA> 24 com_inf - com_uninf 0.338445629 0.5975799 60 0.566360456 5.732612e-01 6.530082e-01

254 IL_21 group_within_dpi <NA> 24 com_inf - sup_inf -0.726966151 0.5975799 60 -1.216517056 2.285520e-01 3.739942e-01

255 IL_21 group_within_dpi <NA> 24 com_inf - sup_uninf 0.332101958 0.5975799 60 0.555744852 5.804518e-01 6.530082e-01

256 IL_21 group_within_dpi <NA> 24 com_uninf - sup_inf -1.065411780 0.5975799 60 -1.782877512 7.966502e-02 1.465705e-01

257 IL_21 group_within_dpi <NA> 24 com_uninf - sup_uninf -0.006343671 0.5975799 60 -0.010615603 9.915654e-01 9.915654e-01

258 IL_21 group_within_dpi <NA> 24 sup_inf - sup_uninf 1.059068109 0.5975799 60 1.772261909 8.142805e-02 1.465705e-01

259 IL_23 group_within_dpi <NA> 8 com_inf - com_uninf -0.984016580 0.5821755 60 -1.690240381 9.617161e-02 2.472984e-01

*260 IL_23 group_within_dpi <NA> 8 com_inf - sup_inf -4.498375814 0.5821755 60 -7.726837741 1.434166e-10 2.581498e-09*

*261 IL_23 group_within_dpi <NA> 8 com_inf - sup_uninf -2.959184209 0.5821755 60 -5.082975984 3.900521e-06 2.340313e-05*

*262 IL_23 group_within_dpi <NA> 8 com_uninf - sup_inf -3.514359234 0.5821755 60 -6.036597359 1.067264e-07 9.605372e-07*

*263 IL_23 group_within_dpi <NA> 8 com_uninf - sup_uninf -1.975167629 0.5821755 60 -3.392735603 1.230476e-03 5.537141e-03*

*264 IL_23 group_within_dpi <NA> 8 sup_inf - sup_uninf 1.539191605 0.5821755 60 2.643861757 1.044389e-02 3.759800e-02*

265 IL_23 group_within_dpi <NA> 16 com_inf - com_uninf -1.071740471 0.5821755 60 -1.840923272 7.057854e-02 2.117356e-01

266 IL_23 group_within_dpi <NA> 16 com_inf - sup_inf -0.925457164 0.5821755 60 -1.589653164 1.171689e-01 2.636301e-01

267 IL_23 group_within_dpi <NA> 16 com_inf - sup_uninf -0.872766174 0.5821755 60 -1.499146112 1.390792e-01 2.781585e-01

268 IL_23 group_within_dpi <NA> 16 com_uninf - sup_inf 0.146283307 0.5821755 60 0.251270108 8.024639e-01 9.296669e-01

269 IL_23 group_within_dpi <NA> 16 com_uninf - sup_uninf 0.198974297 0.5821755 60 0.341777160 7.337128e-01 9.296669e-01

270 IL_23 group_within_dpi <NA> 16 sup_inf - sup_uninf 0.052690990 0.5821755 60 0.090507052 9.281858e-01 9.296669e-01

271 IL_23 group_within_dpi <NA> 24 com_inf - com_uninf 0.051601307 0.5821755 60 0.088635309 9.296669e-01 9.296669e-01

272 IL_23 group_within_dpi <NA> 24 com_inf - sup_inf -0.587127306 0.5821755 60 -1.008505650 3.172617e-01 5.191555e-01

273 IL_23 group_within_dpi <NA> 24 com_inf - sup_uninf -0.475823780 0.5821755 60 -0.817320138 4.169792e-01 5.773558e-01

274 IL_23 group_within_dpi <NA> 24 com_uninf - sup_inf -0.638728613 0.5821755 60 -1.097140959 2.769636e-01 4.985344e-01

275 IL_23 group_within_dpi <NA> 24 com_uninf - sup_uninf -0.527425088 0.5821755 60 -0.905955447 3.685833e-01 5.528749e-01

276 IL_23 group_within_dpi <NA> 24 sup_inf - sup_uninf 0.111303525 0.5821755 60 0.191185512 8.490257e-01 9.296669e-01

*277 INFgamma group_within_dpi <NA> 8 com_inf - com_uninf 2.485577054 0.6580461 60 3.777208229 3.665851e-04 3.299266e-03*

278 INFgamma group_within_dpi <NA> 8 com_inf - sup_inf 1.335418019 0.6580461 60 2.029368561 4.686358e-02 1.468624e-01

*279 INFgamma group_within_dpi <NA> 8 com_inf - sup_uninf 3.641771581 0.6580461 60 5.534219735 7.253501e-07 1.305630e-05*

280 INFgamma group_within_dpi <NA> 8 com_uninf - sup_inf -1.150159035 0.6580461 60 -1.747839668 8.560752e-02 1.926169e-01

281 INFgamma group_within_dpi <NA> 8 com_uninf - sup_uninf 1.156194527 0.6580461 60 1.757011505 8.401752e-02 1.926169e-01

*282 INFgamma group_within_dpi <NA> 8 sup_inf - sup_uninf 2.306353562 0.6580461 60 3.504851173 8.706702e-04 5.224021e-03*

283 INFgamma group_within_dpi <NA> 16 com_inf - com_uninf -0.846750076 0.6580461 60 -1.286764114 2.031191e-01 4.062381e-01

284 INFgamma group_within_dpi <NA> 16 com_inf - sup_inf -0.783019608 0.6580461 60 -1.189916081 2.387665e-01 4.297798e-01

285 INFgamma group_within_dpi <NA> 16 com_inf - sup_uninf 0.539529792 0.6580461 60 0.819896679 4.155202e-01 6.232804e-01

286 INFgamma group_within_dpi <NA> 16 com_uninf - sup_inf 0.063730468 0.6580461 60 0.096848034 9.231699e-01 9.231699e-01

287 INFgamma group_within_dpi <NA> 16 com_uninf - sup_uninf 1.386279868 0.6580461 60 2.106660793 3.933737e-02 1.468624e-01

288 INFgamma group_within_dpi <NA> 16 sup_inf - sup_uninf 1.322549400 0.6580461 60 2.009812759 4.895414e-02 1.468624e-01

289 INFgamma group_within_dpi <NA> 24 com_inf - com_uninf -0.429775127 0.6580461 60 -0.653107955 5.161816e-01 6.636621e-01

290 INFgamma group_within_dpi <NA> 24 com_inf - sup_inf 0.066234421 0.6580461 60 0.100653166 9.201614e-01 9.231699e-01

291 INFgamma group_within_dpi <NA> 24 com_inf - sup_uninf 0.164050139 0.6580461 60 0.249298864 8.039811e-01 9.231699e-01

292 INFgamma group_within_dpi <NA> 24 com_uninf - sup_inf 0.496009548 0.6580461 60 0.753761121 4.539406e-01 6.285331e-01

293 INFgamma group_within_dpi <NA> 24 com_uninf - sup_uninf 0.593825266 0.6580461 60 0.902406819 3.704493e-01 6.061898e-01

294 INFgamma group_within_dpi <NA> 24 sup_inf - sup_uninf 0.097815719 0.6580461 60 0.148645699 8.823317e-01 9.231699e-01

295 TNFa group_within_dpi <NA> 8 com_inf - com_uninf 1.371945567 0.6057054 60 2.265037673 2.713444e-02 6.105249e-02

296 TNFa group_within_dpi <NA> 8 com_inf - sup_inf 0.594980169 0.6057054 60 0.982292980 3.299000e-01 4.241571e-01

*297 TNFa group_within_dpi <NA> 8 com_inf - sup_uninf 1.977103760 0.6057054 60 3.264134241 1.815586e-03 6.536110e-03*

298 TNFa group_within_dpi <NA> 8 com_uninf - sup_inf -0.776965397 0.6057054 60 -1.282744693 2.045148e-01 3.067723e-01

299 TNFa group_within_dpi <NA> 8 com_uninf - sup_uninf 0.605158194 0.6057054 60 0.999096568 3.217603e-01 4.241571e-01

300 TNFa group_within_dpi <NA> 8 sup_inf - sup_uninf 1.382123591 0.6057054 60 2.281841262 2.606043e-02 6.105249e-02

301 TNFa group_within_dpi <NA> 16 com_inf - com_uninf -1.273304920 0.6057054 60 -2.102185162 3.974260e-02 7.153668e-02

302 TNFa group_within_dpi <NA> 16 com_inf - sup_inf -0.987434547 0.6057054 60 -1.630222441 1.082936e-01 1.772077e-01

303 TNFa group_within_dpi <NA> 16 com_inf - sup_uninf 1.334418602 0.6057054 60 2.203081870 3.144018e-02 6.288036e-02

304 TNFa group_within_dpi <NA> 16 com_uninf - sup_inf 0.285870373 0.6057054 60 0.471962722 6.386654e-01 6.955215e-01

*305 TNFa group_within_dpi <NA> 16 com_uninf - sup_uninf 2.607723522 0.6057054 60 4.305267032 6.263659e-05 1.127459e-03*

*306 TNFa group_within_dpi <NA> 16 sup_inf - sup_uninf 2.321853149 0.6057054 60 3.833304311 3.054916e-04 2.749424e-03*

307 TNFa group_within_dpi <NA> 24 com_inf - com_uninf -0.270415486 0.6057054 60 -0.446447205 6.568814e-01 6.955215e-01

308 TNFa group_within_dpi <NA> 24 com_inf - sup_inf -0.404560227 0.6057054 60 -0.667915826 5.067485e-01 6.080982e-01

*309 TNFa group_within_dpi <NA> 24 com_inf - sup_uninf 1.744347908 0.6057054 60 2.879861871 5.508248e-03 1.652474e-02*

310 TNFa group_within_dpi <NA> 24 com_uninf - sup_inf -0.134144741 0.6057054 60 -0.221468621 8.254796e-01 8.254796e-01

*311 TNFa group_within_dpi <NA> 24 com_uninf - sup_uninf 2.014763394 0.6057054 60 3.326309076 1.505896e-03 6.536110e-03*

*312 TNFa group_within_dpi <NA> 24 sup_inf - sup_uninf 2.148908135 0.6057054 60 3.547777697 7.614416e-04 4.568650e-03*

313 MIP_2 group_within_dpi <NA> 8 com_inf - com_uninf 0.281451522 0.5858434 60 0.480421099 6.326750e-01 7.592100e-01

*314 MIP_2 group_within_dpi <NA> 8 com_inf - sup_inf -3.876763929 0.5858434 60 -6.617406708 1.119890e-08 1.007901e-07*

315 MIP_2 group_within_dpi <NA> 8 com_inf - sup_uninf -0.680379928 0.5858434 60 -1.161368292 2.500916e-01 3.462807e-01

*316 MIP_2 group_within_dpi <NA> 8 com_uninf - sup_inf -4.158215451 0.5858434 60 -7.097827807 1.703377e-09 3.066079e-08*

317 MIP_2 group_within_dpi <NA> 8 com_uninf - sup_uninf -0.961831450 0.5858434 60 -1.641789391 1.058655e-01 1.732344e-01

*318 MIP_2 group_within_dpi <NA> 8 sup_inf - sup_uninf 3.196384001 0.5858434 60 5.456038417 9.737042e-07 5.842225e-06*

319 MIP_2 group_within_dpi <NA> 16 com_inf - com_uninf 0.202855947 0.5858434 60 0.346263102 7.303563e-01 7.733185e-01

320 MIP_2 group_within_dpi <NA> 16 com_inf - sup_inf 0.246716387 0.5858434 60 0.421130279 6.751647e-01 7.595603e-01

*321 MIP_2 group_within_dpi <NA> 16 com_inf - sup_uninf 1.675359125 0.5858434 60 2.859738925 5.824557e-03 1.747367e-02*

322 MIP_2 group_within_dpi <NA> 16 com_uninf - sup_inf 0.043860441 0.5858434 60 0.074867178 9.405695e-01 9.405695e-01

*323 MIP_2 group_within_dpi <NA> 16 com_uninf - sup_uninf 1.472503178 0.5858434 60 2.513475823 1.465744e-02 3.769057e-02*

*324 MIP_2 group_within_dpi <NA> 16 sup_inf - sup_uninf 1.428642737 0.5858434 60 2.438608645 1.772065e-02 3.987145e-02*

325 MIP_2 group_within_dpi <NA> 24 com_inf - com_uninf -1.284366375 0.5858434 60 -2.192337429 3.224515e-02 6.449031e-02

326 MIP_2 group_within_dpi <NA> 24 com_inf - sup_inf 0.783867277 0.5858434 60 1.338015074 1.859406e-01 2.789110e-01

327 MIP_2 group_within_dpi <NA> 24 com_inf - sup_uninf 1.188330800 0.5858434 60 2.028410383 4.696419e-02 8.453554e-02

*328 MIP_2 group_within_dpi <NA> 24 com_uninf - sup_inf 2.068233652 0.5858434 60 3.530352503 8.041088e-04 2.894792e-03*

*329 MIP_2 group_within_dpi <NA> 24 com_uninf - sup_uninf 2.472697175 0.5858434 60 4.220747812 8.371333e-05 3.767100e-04*

330 MIP_2 group_within_dpi <NA> 24 sup_inf - sup_uninf 0.404463523 0.5858434 60 0.690395309 4.926078e-01 6.333528e-01

As the MANOVA and GLS showed statistically significant multivariate effects of gene vectors over dpi and among groups, principal component analyses (PCAs) were performed to indicate the relative effects of the eleven genes (Table S5).

**Table S5.** Principal component analysis of all log-fold mRNA data across all days post-infection (dpi). Importance of components shows contributions from each component. top_PC1 shows contributions of genes to all components ordered according to PC1. top_PC2 shows the same data ordered according to PC2.

**Importance of components:**

PC1 PC2 PC3 PC4 PC5 PC6 PC7 PC8 PC9 PC10 PC11

Standard deviation 2.0076 1.3093 1.2194 0.9065 0.88192 0.77414 0.68348 0.64093 0.52913 0.49282 0.41082

Proportion of Variance 0.3664 0.1558 0.1352 0.0747 0.07071 0.05448 0.04247 0.03734 0.02545 0.02208 0.01534

Cumulative Proportion 0.3664 0.5222 0.6574 0.7321 0.80283 0.85731 0.89978 0.93712 0.96258 0.98466 1.00000

**top_PC1**

gene PC1 PC2 PC3 PC4 PC5 PC6 PC7 PC8 PC9 PC10 PC11

1 IL_10 18.6 2.72 0.375 0.242 0.0386 0.111 6.32 1.20 5.85 48.9 15.6

2 TNFa 15.7 0.259 9.74 2.79 2.16 1.95 0.545 19.4 5.18 0.473 41.9

3 MIP_2 14.1 3.64 9.03 1.51 3.12 1.20 0.155 6.24 43.1 17.9 0.0199

4 NLRP3 13.6 9.00 0.385 6.39 1.29 4.06 1.07 28.8 20.4 0.429 14.7

5 IL_17A 11.3 4.58 1.29 0.481 40.9 3.09 5.95 6.08 0.949 8.80 16.6

6 INFgamma 10.8 0.000187 7.77 7.60 16.3 1.64 51.1 0.198 3.28 0.0562 1.30

7 IL_21 6.49 8.77 24.9 0.000246 3.39 0.0241 25.8 0.233 6.14 15.4 8.82

8 IL_23 5.57 1.17 21.7 15.0 19.0 11.5 0.0142 15.8 9.40 0.724 0.173

9 IL_18 2.14 0.400 23.0 62.8 0.243 0.642 7.38 0.0733 3.01 0.339 0.0472

10 IL_1B 0.943 32.9 1.82 1.19 7.09 44.7 0.342 5.47 1.99 3.40 0.162

11 PTGS2 0.805 36.6 0.0298 2.05 6.58 31.1 1.29 16.5 0.776 3.55 0.737

**top_PC2**

gene PC1 PC2 PC3 PC4 PC5 PC6 PC7 PC8 PC9 PC10 PC11

1 PTGS2 0.805 36.6 0.0298 2.05 6.58 31.1 1.29 16.5 0.776 3.55 0.737

2 IL_1B 0.943 32.9 1.82 1.19 7.09 44.7 0.342 5.47 1.99 3.40 0.162

3 NLRP3 13.6 9.00 0.385 6.39 1.29 4.06 1.07 28.8 20.4 0.429 14.7

4 IL_21 6.49 8.77 24.9 0.000246 3.39 0.0241 25.8 0.233 6.14 15.4 8.82

5 IL_17A 11.3 4.58 1.29 0.481 40.9 3.09 5.95 6.08 0.949 8.80 16.6

6 MIP_2 14.1 3.64 9.03 1.51 3.12 1.20 0.155 6.24 43.1 17.9 0.0199

7 IL_10 18.6 2.72 0.375 0.242 0.0386 0.111 6.32 1.20 5.85 48.9 15.6

8 IL_23 5.57 1.17 21.7 15.0 19.0 11.5 0.0142 15.8 9.40 0.724 0.173

9 IL_18 2.14 0.400 23.0 62.8 0.243 0.642 7.38 0.0733 3.01 0.339 0.0472

10 TNFa 15.7 0.259 9.74 2.79 2.16 1.95 0.545 19.4 5.18 0.473 41.9

11 INFgamma 10.8 0.000187 7.77 7.60 16.3 1.64 51.1 0.198 3.28 0.0562 1.30

However, it was decided to analyze the dpi separately, as shown in Table S6.

**Table S6.** Principal component analysis of all log-fold mRNA data at either A) days post-infection (dpi) 8; B) dpi 16; or C) dpi 24. Importance of components shows contributions from each component. top_PC1 shows contributions of genes to all components ordered according to PC1. top_PC2 shows the same data ordered according to PC2.

**A) DPI 8.**

Importance of components:

PC1 PC2 PC3 PC4 PC5 PC6 PC7

Standard deviation 2.0614 1.6323 1.1863 0.94769 0.68642 0.6724 0.52783

Proportion of Variance 0.3863 0.2422 0.1279 0.08165 0.04283 0.0411 0.02533

Cumulative Proportion 0.3863 0.6286 0.7565 0.83813 0.88096 0.9221 0.94739

PC8 PC9 PC10 PC11

Standard deviation 0.47434 0.39913 0.34344 0.27643

Proportion of Variance 0.02045 0.01448 0.01072 0.00695

Cumulative Proportion 0.96785 0.98233 0.99305 1.00000

**top_PC1_8**

gene PC1 PC2 PC3 PC4 PC5 PC6

1 IL_10 18.807734 3.877378e-01 1.530546e+00 0.8213190 0.2495873 0.7258402

2 MIP_2 13.132323 5.396769e+00 9.028800e+00 3.7354210 2.3333086 7.6755427

3 NLRP3 11.073438 1.557185e-02 2.415193e+01 0.5230459 4.5887296 26.1652507

4 IL_1B 10.088208 3.611697e+00 1.333163e-04 39.4687539 10.5069917 0.0546997

5 TNFa 8.871974 1.533030e+01 4.756644e-04 3.9327575 4.4412474 24.2393782

6 IL_17A 8.138838 3.148766e-04 2.938186e+01 14.8871351 1.5544849 5.6776085

7 INFgamma 8.107947 1.497523e+01 2.278632e+00 0.1028497 26.1697780 9.2726462

8 IL_21 6.949866 1.510480e+01 6.007046e+00 0.9816487 15.4967880 3.2365957

9 IL_23 6.510607 2.216352e+01 1.891893e+00 0.1377531 7.8025614 1.2354137

10 PTGS2 6.293637 6.809396e-01 2.546154e+01 24.9098365 0.9818255 19.4891924

11 IL_18 2.025428 2.233313e+01 2.671373e-01 10.4994795 25.8746975 2.2278321

PC7 PC8 PC9 PC10 PC11

1 0.267156966 52.6690800 21.9883592 1.9787799 0.5738597

2 16.727709148 0.7515528 10.5544248 7.9324630 22.7316868

3 3.802041294 1.0288526 8.8497208 5.7574491 14.0439668

4 0.001062969 21.8557598 12.7496969 0.1671854 1.4958108

5 0.032308348 0.1648333 1.0861878 34.7723087 7.1282332

6 11.327695848 10.1159946 0.7024642 11.0917991 7.1218021

7 4.346379856 0.7865864 21.7374074 0.8703197 11.3522286

8 34.484580091 4.3319211 1.4317020 9.4761267 2.4989297

9 0.019659949 1.1572095 6.3937570 22.7620421 29.9255825

10 0.770221989 6.8979969 13.0890317 0.0946769 1.3311011

11 28.221183543 0.2402128 1.4172481 5.0968495 1.7967988

**top_PC2_8**

gene PC1 PC2 PC3 PC4 PC5 PC6

1 IL_18 2.025428 2.233313e+01 2.671373e-01 10.4994795 25.8746975 2.2278321

2 IL_23 6.510607 2.216352e+01 1.891893e+00 0.1377531 7.8025614 1.2354137

3 TNFa 8.871974 1.533030e+01 4.756644e-04 3.9327575 4.4412474 24.2393782

4 IL_21 6.949866 1.510480e+01 6.007046e+00 0.9816487 15.4967880 3.2365957

5 INFgamma 8.107947 1.497523e+01 2.278632e+00 0.1028497 26.1697780 9.2726462

6 MIP_2 13.132323 5.396769e+00 9.028800e+00 3.7354210 2.3333086 7.6755427

7 IL_1B 10.088208 3.611697e+00 1.333163e-04 39.4687539 10.5069917 0.0546997

8 PTGS2 6.293637 6.809396e-01 2.546154e+01 24.9098365 0.9818255 19.4891924

9 IL_10 18.807734 3.877378e-01 1.530546e+00 0.8213190 0.2495873 0.7258402

10 NLRP3 11.073438 1.557185e-02 2.415193e+01 0.5230459 4.5887296 26.1652507

11 IL_17A 8.138838 3.148766e-04 2.938186e+01 14.8871351 1.5544849 5.6776085

PC7 PC8 PC9 PC10 PC11

1 28.221183543 0.2402128 1.4172481 5.0968495 1.7967988

2 0.019659949 1.1572095 6.3937570 22.7620421 29.9255825

3 0.032308348 0.1648333 1.0861878 34.7723087 7.1282332

4 34.484580091 4.3319211 1.4317020 9.4761267 2.4989297

5 4.346379856 0.7865864 21.7374074 0.8703197 11.3522286

6 16.727709148 0.7515528 10.5544248 7.9324630 22.7316868

7 0.001062969 21.8557598 12.7496969 0.1671854 1.4958108

8 0.770221989 6.8979969 13.0890317 0.0946769 1.3311011

9 0.267156966 52.6690800 21.9883592 1.9787799 0.5738597

10 3.802041294 1.0288526 8.8497208 5.7574491 14.0439668

11 11.327695848 10.1159946 0.7024642 11.0917991 7.1218021

**B. DPI 16.**

Importance of components:

PC1 PC2 PC3 PC4 PC5 PC6 PC7

Standard deviation 2.0958 1.5569 1.1972 0.93226 0.89809 0.6222 0.54610

Proportion of Variance 0.3993 0.2204 0.1303 0.07901 0.07332 0.0352 0.02711

Cumulative Proportion 0.3993 0.6197 0.7500 0.82898 0.90230 0.9375 0.96461

PC8 PC9 PC10 PC11

Standard deviation 0.43687 0.33865 0.27105 0.10164

Proportion of Variance 0.01735 0.01043 0.00668 0.00094

Cumulative Proportion 0.98196 0.99238 0.99906 1.00000

**top_PC1_16**

gene PC1 PC2 PC3 PC4 PC5 PC6

1 TNFa 19.1779748 0.8176399 0.07094639 3.8388290 7.1707729 4.622199e-01

2 IL_10 18.8291915 0.9597098 0.59506194 5.2254347 3.6865430 3.615482e+00

3 NLRP3 14.5470266 4.6028788 0.59190634 7.4054700 7.8102476 4.369808e-04

4 INFgamma 14.2503316 1.2347145 9.34507605 8.1906055 1.9344196 1.259332e+01

5 MIP_2 12.5348902 6.1412949 4.90924400 0.8598458 10.0941365 1.889191e+01

6 IL_21 9.7063398 7.4225120 8.03117894 19.0068164 0.3059330 4.995014e+00

7 IL_1B 4.6000725 27.0251618 0.73774340 1.8633336 4.3412965 1.735922e+00

8 IL_23 2.9013804 13.1243829 4.00513415 9.4624025 36.6577083 2.587391e+01

9 IL_17A 2.1835764 3.3325050 46.52082006 7.0284947 5.8717769 1.752879e+00

10 IL_18 0.8867104 5.9934810 20.77793015 34.2703043 21.8212980 2.793300e-01

11 PTGS2 0.3825057 29.3457193 4.41495858 2.8484635 0.3058677 2.979958e+01

PC7 PC8 PC9 PC10 PC11

1 7.914185 7.680471 0.1006351 0.007674482 52.75865162

2 5.927099 15.081458 0.4335164 0.580685389 45.06581862

3 26.768049 3.496118 3.8569043 30.919564892 0.00139733

4 14.116557 3.399041 1.1404828 33.720142068 0.07530657

5 7.780966 5.382762 24.7747334 8.543868825 0.08635027

6 1.035066 44.431155 0.2189669 4.756533017 0.09048457

7 2.256496 2.335273 55.0601033 0.006743823 0.03785367

8 5.095921 1.436155 0.5095061 0.501761132 0.43174213

9 3.650739 7.588331 0.4871033 20.802818706 0.78095581

10 12.693045 2.053258 1.0499804 0.043200384 0.13146237

11 12.761877 7.115977 12.3680681 0.117007281 0.53997704

**top_PC2_16**

gene PC1 PC2 PC3 PC4 PC5 PC6

1 PTGS2 0.3825057 29.3457193 4.41495858 2.8484635 0.3058677 2.979958e+01

2 IL_1B 4.6000725 27.0251618 0.73774340 1.8633336 4.3412965 1.735922e+00

3 IL_23 2.9013804 13.1243829 4.00513415 9.4624025 36.6577083 2.587391e+01

4 IL_21 9.7063398 7.4225120 8.03117894 19.0068164 0.3059330 4.995014e+00

5 MIP_2 12.5348902 6.1412949 4.90924400 0.8598458 10.0941365 1.889191e+01

6 IL_18 0.8867104 5.9934810 20.77793015 34.2703043 21.8212980 2.793300e-01

7 NLRP3 14.5470266 4.6028788 0.59190634 7.4054700 7.8102476 4.369808e-04

8 IL_17A 2.1835764 3.3325050 46.52082006 7.0284947 5.8717769 1.752879e+00

9 INFgamma 14.2503316 1.2347145 9.34507605 8.1906055 1.9344196 1.259332e+01

10 IL_10 18.8291915 0.9597098 0.59506194 5.2254347 3.6865430 3.615482e+00

11 TNFa 19.1779748 0.8176399 0.07094639 3.8388290 7.1707729 4.622199e-01

PC7 PC8 PC9 PC10 PC11

1 12.761877 7.115977 12.3680681 0.117007281 0.53997704

2 2.256496 2.335273 55.0601033 0.006743823 0.03785367

3 5.095921 1.436155 0.5095061 0.501761132 0.43174213

4 1.035066 44.431155 0.2189669 4.756533017 0.09048457

5 7.780966 5.382762 24.7747334 8.543868825 0.08635027

6 12.693045 2.053258 1.0499804 0.043200384 0.13146237

7 26.768049 3.496118 3.8569043 30.919564892 0.00139733

8 3.650739 7.588331 0.4871033 20.802818706 0.78095581

9 14.116557 3.399041 1.1404828 33.720142068 0.07530657

10 5.927099 15.081458 0.4335164 0.580685389 45.06581862

11 7.914185 7.680471 0.1006351 0.007674482 52.75865162

**C. DPI 24.**

Importance of components:

PC1 PC2 PC3 PC4 PC5 PC6 PC7 PC8

Standard deviation 1.8809 1.4768 1.2966 1.0898 0.78112 0.67774 0.6708 0.6034

Proportion of Variance 0.3216 0.1983 0.1528 0.1080 0.05547 0.04176 0.0409 0.0331

Cumulative Proportion 0.3216 0.5199 0.6727 0.7807 0.83617 0.87793 0.9188 0.9519

PC9 PC10 PC11

Standard deviation 0.53367 0.4048 0.28288

Proportion of Variance 0.02589 0.0149 0.00727

Cumulative Proportion 0.97783 0.9927 1.00000

**top_PC1_24**

gene PC1 PC2 PC3 PC4 PC5

1 IL_10 21.4359147 0.6722412 0.05615228 0.02665985 1.336821e+00

2 IL_17A 19.2968499 1.7373965 2.18688686 2.55020512 9.969506e+00

3 MIP_2 17.7038823 3.6881096 5.38719110 0.13487323 1.622526e+00

4 TNFa 17.4367879 8.1948514 0.01681456 0.03444219 1.412877e-02

5 NLRP3 12.0532000 8.3406987 0.52409027 18.39447081 8.138234e-01

6 INFgamma 8.5717925 0.3666560 10.89309607 28.22633684 6.057242e+00

7 IL_23 1.3280508 11.1282069 19.38561058 5.38640912 3.583580e+01

8 IL_21 0.9350758 23.6465256 4.61241550 8.19464903 2.037121e+01

9 IL_1B 0.8706070 15.3433658 21.40122260 0.84425847 1.378616e+01

10 IL_18 0.1859374 0.5381133 24.48752538 36.16985699 7.037455e-04

11 PTGS2 0.1819018 26.3438349 11.04899479 0.03783836 1.019208e+01

PC6 PC7 PC8 PC9 PC10 PC11

1 0.05023454 5.637380613 45.32039408 1.0633700 5.18340952 19.2174227

2 16.10811718 4.395101023 0.02363641 10.5103985 1.36506822 31.8568341

3 3.05533453 0.009388121 10.88235748 36.5938244 19.64957449 1.2729383

4 8.08949105 14.347207111 18.79099516 2.6142169 1.94341677 28.5176482

5 14.28900633 6.046751670 1.36663042 0.2811863 36.32766398 1.5624780

6 0.04000855 19.240654854 1.64080977 1.1383809 21.90818836 1.9168340

7 6.22184833 8.479966828 4.43028587 5.4935915 1.71182069 0.5984119

8 24.23561968 0.007714739 1.13408757 9.1882961 5.04175308 2.6326549

9 9.24237466 26.548892733 1.71292675 1.1255738 2.38283075 6.7417884

10 11.29760660 8.873485942 0.18884406 16.2433072 0.06421293 1.9504064

11 7.37035855 6.413456365 14.50903243 15.7478542 4.42206122 3.7325831

**top_PC2_24**

gene PC1 PC2 PC3 PC4 PC5

1 PTGS2 0.1819018 26.3438349 11.04899479 0.03783836 1.019208e+01

2 IL_21 0.9350758 23.6465256 4.61241550 8.19464903 2.037121e+01

3 IL_1B 0.8706070 15.3433658 21.40122260 0.84425847 1.378616e+01

4 IL_23 1.3280508 11.1282069 19.38561058 5.38640912 3.583580e+01

5 NLRP3 12.0532000 8.3406987 0.52409027 18.39447081 8.138234e-01

6 TNFa 17.4367879 8.1948514 0.01681456 0.03444219 1.412877e-02

7 MIP_2 17.7038823 3.6881096 5.38719110 0.13487323 1.622526e+00

8 IL_17A 19.2968499 1.7373965 2.18688686 2.55020512 9.969506e+00

9 IL_10 21.4359147 0.6722412 0.05615228 0.02665985 1.336821e+00

10 IL_18 0.1859374 0.5381133 24.48752538 36.16985699 7.037455e-04

11 INFgamma 8.5717925 0.3666560 10.89309607 28.22633684 6.057242e+00

PC6 PC7 PC8 PC9 PC10 PC11

1 7.37035855 6.413456365 14.50903243 15.7478542 4.42206122 3.7325831

2 24.23561968 0.007714739 1.13408757 9.1882961 5.04175308 2.6326549

3 9.24237466 26.548892733 1.71292675 1.1255738 2.38283075 6.7417884

4 6.22184833 8.479966828 4.43028587 5.4935915 1.71182069 0.5984119

5 14.28900633 6.046751670 1.36663042 0.2811863 36.32766398 1.5624780

6 8.08949105 14.347207111 18.79099516 2.6142169 1.94341677 28.5176482

7 3.05533453 0.009388121 10.88235748 36.5938244 19.64957449 1.2729383

8 16.10811718 4.395101023 0.02363641 10.5103985 1.36506822 31.8568341

9 0.05023454 5.637380613 45.32039408 1.0633700 5.18340952 19.2174227

10 11.29760660 8.873485942 0.18884406 16.2433072 0.06421293 1.9504064

11 0.04000855 19.240654854 1.64080977 1.1383809 21.90818836 1.9168340
