# Supplementary material for: Immunological and inflammatory responses in the kidneys in experimental acanthamoebiasis
Source: Microbiol Spectr. 2025 Jun 30;13(8):e00243-25. doi: 10.1128/spectrum.00243-25 (PMC12323576; doi:10.1128/spectrum.00243-25)
Supplement: Supplemental File S2 — Code for PCA in the R statistical platform. [file spectrum.00243-25-s0002.docx]

**Title: Code - PCA**

**setwd()**

mypath <- getwd()

mypathMain <- paste0(mypath, "/", "Main", "/")

##

if (!dir.exists(mypathMain)) {

suppressWarnings(dir.create(mypathMain))

}

**## libraries and accessories:**

options(timeout=1000)

ipak <- function(pkg){

new.pkg <- pkg[!(pkg %in% installed.packages()[, "Package"])]

if (length(new.pkg))

install.packages(new.pkg, dependencies = TRUE, repos="http://cran.r-project.org")

sapply(pkg, require, character.only = TRUE)

} ## end function

packages <- c("data.table", "ggbiplot", "ggrepel", "emmeans", "car", "RVAideMemoire", "lmtest", "sandwich", "ggplot2", "dplyr", "tibble", "janitor", "tidyr", "purrr", "broom", "broom.mixed", "modelr", "nlme")

ipak(packages)

**FILENAME_pca_2 <- "pca_2" ## do not put .csv**

**studyname <- "Kot2025"**

**substudy <- "main"**

cbp2 <- c("#D55E00", "#56B4E9", "#009E73", "#E69F00", "#F0E442", "#0072B2", "#CC79A7", "#000000") ## color-blind with black

scale_colour_discrete <- function(...) {

ggplot2::scale_colour_manual(values = cbp2, ...)

}

scale_fill_discrete <- function(...) {

ggplot2::scale_fill_manual(values = cbp2, ...)

}

theme_jack <- function (base_size = 16, font = "Helvetica", base_family = "", axisColor='#999999', textColor='black') {

theme_classic(base_size = base_size, base_family = base_family) %+replace%

theme(

plot.title = element_text(size=15, vjust=3),

axis.text.x = element_text(colour = "black", family="Times", face=c('bold'), size = 18, vjust = 0.2),

axis.text.y = element_text(colour = "black", family="Times", face=c('bold'), size = 18),

axis.title.x = element_text(colour = "black", family="Times", face=c('bold'), vjust = -4, size = 18),

axis.title.y = element_text(colour = "black", family="Times", angle=90, face=c('bold'), vjust= 5, size = 18),

panel.grid.minor = element_blank(),

panel.grid.major = element_blank(),

plot.background = element_rect(fill="white"),

panel.border = element_blank(),

panel.background = element_blank(),

plot.margin=unit(c(1,1.5,1.3,1.3),"cm")

)

}

theme_set(theme_jack())

newSlash <- stringr::str_replace_all(normalizePath("/"), "C:", "")

newSlash

mypathname <- paste0(getwd(), newSlash) ## for R file only

mypathnameSUB <- paste0(mypathname, substudy, newSlash)

**## create SUB-STUDY FOLDER to put Folders into**

substudydirectory <- paste0(mypathname, substudy)

if (!dir.exists(substudydirectory)) {

suppressWarnings(dir.create(substudydirectory))

}

**##** To create **FOLDER NAMEs** inside substudy folder:

graphsFolder <- paste0(studyname, "_", substudy, "_Graphs")

graphsFolder

graphsFolderdirectory <- paste0(mypathnameSUB, graphsFolder)

if (!dir.exists(graphsFolderdirectory)) {

suppressWarnings(dir.create(graphsFolderdirectory))

}

corrGraphsFolder <- paste0(studyname, "_", substudy, "_CorrGraphs")

corrGraphsFolder

corrGraphsFolderdirectory <- paste0(mypathnameSUB, corrGraphsFolder)

if (!dir.exists(corrGraphsFolderdirectory)) {

suppressWarnings(dir.create(corrGraphsFolderdirectory))

}

corrGraphsNumFolder <- paste0(studyname, "_", substudy, "_CorrGraphsNum")

corrGraphsNumFolder

corrGraphsNumFolderdirectory <- paste0(mypathnameSUB, corrGraphsNumFolder)

if (!dir.exists(corrGraphsNumFolderdirectory)) {

suppressWarnings(dir.create(corrGraphsNumFolderdirectory))

}

reportsFolder <- paste0(studyname, "_", substudy, "_REPORTS")

reportsFolder

reportsFolderdirectory <- paste0(mypathnameSUB, reportsFolder)

if (!dir.exists(reportsFolderdirectory)) {

suppressWarnings(dir.create(reportsFolderdirectory))

}

lm_graphsFolder <- paste0(studyname, "_", substudy, "_lm_graphs")

lm_graphsFolder

lm_graphsFolderdirectory <- paste0(mypathnameSUB, lm_graphsFolder)

if (!dir.exists(lm_graphsFolderdirectory)) {

suppressWarnings(dir.create(lm_graphsFolderdirectory))

}

log_graphsFolder <- paste0(studyname, "_", substudy, "_logistic_graphs")

log_graphsFolder

log_graphsFolderdirectory <- paste0(mypathnameSUB, log_graphsFolder)

if (!dir.exists(log_graphsFolderdirectory)) {

suppressWarnings(dir.create(log_graphsFolderdirectory))

}

mRNA <- data.table::fread(file = paste0(mypathname, FILENAME_pca_2, ".csv"), encoding = "UTF-8")

mRNA <- as.data.frame(mRNA)

mRNA$dpi <- as.factor(mRNA$dpi)

mRNA$group <- as.factor(mRNA$group)

class(mRNA)

lapply(mRNA, class)

## the numbers can be relativised again, by difference between natural logarithm of each number minus the mean natural logarithm for time 8 uninfected immunocompetent:

logdf <- mRNA

for (i in 7:ncol(logdf)) {

for (j in 1:nrow(logdf)) {

logdf[j, i] <- log(mRNA[j, i])

}
}
head(logdf)

## to get mean natural logarithm for time 8 uninfected immunocompetent:

comp_uninf_df <- logdf %>% ## add means

janitor::adorn_totals(name = "mean") %>% mutate(across(where(is.numeric), ~ replace(., n(), .[n()]/(n()-1))))

colnames(comp_uninf_df)

comp_uninf_means2 <- comp_uninf_df[comp_uninf_df$Mouse_ID == "mean", ]

comp_uninf_means <- as.vector(unlist(comp_uninf_means2))

comp_uninf_means <- as.numeric(comp_uninf_means)

names(comp_uninf_means) <- colnames(comp_uninf_means2)

comp_uninf_means

relatdf <- logdf

for (i in 7:ncol(relatdf)) {

for (j in 1:nrow(relatdf)) {

relatdf[j, i] <- logdf[j, i] - comp_uninf_means[i]

}
}
relatdf

relatdf[37, ]

relatdf[38, ]

relatdf[39, ]

relatdf[40, ]

relatdf[41, ]

**## For residuals plots:**

nocomp <- c("Mouse_ID", "Group_dpi_ID", "immunological_status", "acanthamoeba_infection", "group", "dpi")

resid_df <- relatdf

resid_df <- relatdf %>%

mutate(across(-all_of(nocomp), ~ resid(lm(.x ~ dpi, data = relatdf)),

.names = "{.col}_resid"

)

)

resid_long <- resid_df %>%

pivot_longer(cols = ends_with("_resid"), names_to = "parameter",

values_to = "resid") %>% mutate(parameter = sub("_resid$", "", parameter))

p <- ggplot(resid_long, aes(x = dpi, y = resid)) +

geom_point(alpha = 0.6) +

geom_smooth(method = "loess", se = FALSE,

span = 0.8, linewidth = 0.6) +

facet_wrap(~ parameter, scales = "free_y") +

theme_bw() +

labs(

x = "Days post-infection (dpi)",

y = "Residual (log ratio)",

title = "Time-dependent residuals for each mRNA parameter"

)

print(p)

**## Residuals vs fitted:**

resid_fit_long <- relatdf %>%

pivot_longer(cols = -all_of(nocomp),

names_to = "parameter",

values_to = "y"

) %>%

group_by(parameter) %>%

nest() %>%

mutate(

model = map(data, ~ lm(y ~ dpi, data = .x)),

aug = map2(model, data, ~ augment(.x, data = .y))) %>%

select(parameter, aug) %>%

unnest(aug)

dev.new()

ggplot(resid_fit_long, aes(x = .fitted, y = .resid)) +

geom_point() +

geom_hline(yintercept = 0, linetype = "dashed") +

facet_wrap(~ parameter, scales = "free") +

labs(

x = "Fitted value",

y = "Residual",

title = "Residual vs. fitted for each mRNA parameter"

) +

theme_bw()

## QQ plots:

ggplot(resid_long, aes(sample = resid)) + stat_qq() + stat_qq_line() + facet_wrap(~parameter)

car::leveneTest(resid ~ dpi, data = resid_long)

## The Levene's test for heteroscedasticity was significant, so weights were used in subsequent gls: weights = nlme::varIdent() with generalised least squares.

**## FULL MANOVA MODEL - uses manova, then gls for gene effects**

relatdf <- relatdf %>%

mutate(

dpi = factor(dpi),

group = factor(group)

)

gene_mat <- relatdf %>% select(-all_of(nocomp)) %>% as.matrix()

man2 <- stats::manova(gene_mat ~ dpi * group, data = relatdf)

# Pillai omnibus test - shown in **Table S1.**

summary(man2, test = "Pillai")

gene_cols <- setdiff(names(relatdf), nocomp)

gls_tbl_full <- relatdf %>%

pivot_longer(all_of(gene_cols), names_to = "gene", values_to = "y") %>%

group_by(gene) %>%

nest() %>%

mutate(

gls = suppressWarnings(map(data, ~

nlme::gls(

y ~ dpi * group,

data = .x,

weights = nlme::varIdent(~ 1 | interaction(dpi, group)))

)

)

)

**## OMNIBUS over all groups and all dpi:**

## dpi (averaged over all 4 groups) and group (averaged over all 3 dpi levels) - p.value is the joint Wald test from emmeans::joint_tests(), which uses the GLS heteroscedastic SEs.

main_tests <- gls_tbl_full %>%

transmute(

gene,

jt = map2(gls, data, ~ {

emm <- emmeans(.x, specs = ~ dpi + group, data = .y)

as_tibble(joint_tests(emm))

})

) %>%

unnest(jt) %>%

rename(term = "model term") %>%

filter(term %in% c("dpi", "group")) %>% select(gene,

effect = term,

df1, df2,

F = F.ratio,

p.value) %>%

mutate(p_adj = p.adjust(p.value, method = "BH"))

main_tests2 <- as.data.frame(main_tests)

main_tests2 ## shown in **Table S2.**

**## OMNIBUS over all groups but not dpi:**

## emmeans::joint_tests() gives a multivariate Wald-F test.

##

**## dpi effect within each group:**

dpi_joint <- gls_tbl_full %>%

transmute(

gene,

jt = map2(gls, data, ~ {

emm <- emmeans(.x, ~ dpi | group, data = .y)

as_tibble(

as.data.frame(joint_tests(emm, by = "group"), separate = TRUE)

)

})

) %>%

unnest(jt) %>%

rename(by = group) %>%

select(gene, by,

F = F.ratio,

df1 = df1,

df2 = df2,

p.value) %>%

mutate(test = "dpi_within_group")

**## group effect within each dpi:**

grp_joint <- gls_tbl_full %>%

transmute(

gene,

jt = map2(gls, data, ~ {

emm <- emmeans(.x, ~ group | dpi, data = .y)

as_tibble(

as.data.frame(joint_tests(emm, by = "dpi"), separate = TRUE)

)

})

) %>%

unnest(jt) %>%

rename(by = dpi) %>%

select(gene, by,

F = F.ratio,

df1 = df1,

df2 = df2,

p.value) %>%

mutate(test = "group_within_dpi")

simple_effects_tbl <- bind_rows(dpi_joint, grp_joint) %>%

mutate(p_adj = p.adjust(p.value, method = "BH"))

simple_effects_tbl2 <- as.data.frame(simple_effects_tbl)

simple_effects_tbl2 ## shown in **Table S3.**

**## ALL CONTRASTS IN FULL MODEL:**

**## dpi contrasts within each group:**

dpi_contrasts <- gls_tbl_full %>%

transmute(

gene,

contr = map2(gls, data, ~ {

emm <- emmeans(.x, ~ dpi | group, data = .y)

as_tibble(as.data.frame(pairs(emm, adjust = "none"), separate = TRUE))

})

) %>%

unnest(contr) %>%

mutate(

contrast_type = "dpi_within_group",

p_adj = p.adjust(p.value, method = "BH")

)

**## group contrasts within each dpi:**

group_contrasts <- gls_tbl_full %>%

transmute(

gene,

contr = map2(gls, data, ~ {

emm <- emmeans(.x, ~ group | dpi, data = .y)

as_tibble(as.data.frame(pairs(emm, adjust = "none"), separate = TRUE))

})

) %>%

unnest(contr) %>%

mutate(

contrast_type = "group_within_dpi",

p_adj = p.adjust(p.value, method = "BH")

)

pair_tbl <- bind_rows(dpi_contrasts, group_contrasts) %>%

select(gene, contrast_type,

group, dpi,

contrast, estimate, SE, df, t.ratio, p.value, p_adj)

pair_tbl2 <- as.data.frame(pair_tbl)

pair_tbl2 ## shown in **Table S4.**

**## Principal component analysis: ALL DATA**

pca_obj <- prcomp(gene_mat, center = TRUE, scale. = TRUE)

summary(pca_obj)

load_tbl <- as_tibble(pca_obj$rotation, rownames = "gene")

## % contributions:

contrib_tbl <- load_tbl %>% mutate(across(starts_with("PC"), ~ (.x^2) * 100))

top_PC1 <- contrib_tbl %>% arrange(desc(PC1))

top_PC2 <- contrib_tbl %>% arrange(desc(PC2))

scores <- as_tibble(pca_obj$x[, 1:2]) # PC1, PC2

relat_pca <- bind_cols(relatdf, scores)

dev.new()

myggplot <- ggplot(relat_pca, aes(PC1, PC2, colour = group, shape = dpi)) +

geom_point(size = 2) +

stat_ellipse(aes(group = interaction(dpi, group)), linetype = "dashed") +

theme_bw()

myggplot

ggsave(paste0("PCA_ggplot_group_dpi_ellipse_alldata", ".pdf"), plot = myggplot, device = "pdf", path = graphsFolderdirectory, width = 8, height = 6, dpi = 300, units = "in")

dev.new()

graphnolabels <- ggbiplot::ggbiplot(pca_obj)

graphnolabels

ggsave(paste0("ggbiplot_nolabels_alldata", ".pdf"), plot = graphnolabels, device = "pdf", path = graphsFolderdirectory, width = 8, height = 6, dpi = 300, units = "in")

dev.new()

graphlabels <- ggbiplot(pca_obj, labels = relatdf$Group_dpi_ID)

graphlabels

ggsave(paste0("ggbiplot_labels_alldata", ".pdf"), plot = graphlabels, device = "pdf", path = graphsFolderdirectory, width = 8, height = 6, dpi = 300, units = "in")

dev.new()

group_ellipse <- ggbiplot(pca_obj, ellipse=TRUE, labels = relatdf$Group_dpi_ID, groups = relatdf$group)

group_ellipse

ggsave(paste0("PCA_group_ellipse_alldata", ".pdf"), plot = group_ellipse, device = "pdf", path = graphsFolderdirectory, width = 8, height = 6, dpi = 300, units = "in")

dev.new()

dpi_ellipse <- ggbiplot(pca_obj, ellipse=TRUE, labels = relatdf$Group_dpi_ID, groups = relatdf$dpi)

dpi_ellipse

ggsave(paste0("PCA_dpi_ellipse_alldata", ".pdf"), plot = dpi_ellipse, device = "pdf", path = graphsFolderdirectory, width = 8, height = 6, dpi = 300, units = "in")

## linear models of PC1 and PC2:

library(lmtest)

lm_PC1 <- lm(PC1 ~ dpi * group, data = relat_pca)

anova(lm_PC1)

## Analysis of Variance Table

##

## Response: PC1

## Df Sum Sq Mean Sq F value Pr(>F)

## dpi 2 59.161 29.5804 17.540 1.004e-06 ***

## group 3 83.924 27.9748 16.588 5.803e-08 ***

## dpi:group 6 41.892 6.9821 4.140 0.001522 **

## Residuals 60 101.188 1.6865

## ---

## Signif. codes: 0 ‘***’ 0.001 ‘**’ 0.01 ‘*’ 0.05 ‘.’ 0.1 ‘ ’ 1

library(lmtest)

lm_PC2 <- lm(PC2 ~ dpi * group, data = relat_pca)

anova(lm_PC2)

## Analysis of Variance Table

##

## Response: PC2

## Df Sum Sq Mean Sq F value Pr(>F)

## dpi 2 13.815 6.9076 12.0556 3.971e-05 ***

## group 3 47.423 15.8076 27.5882 2.449e-11 ***

## dpi:group 6 26.093 4.3489 7.5898 4.410e-06 ***

## Residuals 60 34.379 0.5730

## ---

## Signif. codes: 0 ‘***’ 0.001 ‘**’ 0.01 ‘*’ 0.05 ‘.’ 0.1 ‘ ’ 1

##

## dpi, group and dpi:group interaction are all significant for both PC1 and PC2. We can assume that genes which load strongly into these are at least partly responsible for the MANOVA findings.

summary(pca_obj) ## shown in **Table S5.**

print(top_PC1)

print(top_PC2)

**## Principal component analysis: WITH DPI 8 ONLY**

relatdf8 <- relatdf[relatdf$dpi == 8, ]

gene_mat8 <- relatdf8 %>% select(-all_of(nocomp)) %>% as.matrix()

pca_obj8 <- prcomp(gene_mat8, center = TRUE, scale. = TRUE)

summary(pca_obj8)

load_tbl8 <- as_tibble(pca_obj8$rotation, rownames = "gene")

## squared loading × 100 gives % contribution

contrib_tbl8 <- load_tbl8 %>% mutate(across(starts_with("PC"), ~ (.x^2) * 100))

top_PC1_8 <- contrib_tbl8 %>% arrange(desc(PC1)) %>% as.data.frame()

top_PC2_8 <- contrib_tbl8 %>% arrange(desc(PC2)) %>% as.data.frame()

top_PC1_8

top_PC2_8

scores8 <- as_tibble(pca_obj8$x[, 1:2]) # PC1, PC2

relat_pca8 <- bind_cols(relatdf8, scores8)

dev.new()

graphnolabels8 <- ggbiplot::ggbiplot(pca_obj8)

graphnolabels8

ggsave(paste0("ggbiplot_nolabels_8", ".pdf"), plot = graphnolabels8, device = "pdf", path = graphsFolderdirectory, width = 8, height = 6, dpi = 300, units = "in")

dev.new()

graphlabels8 <- ggbiplot(pca_obj8, labels = relatdf8$Group_dpi_ID)

graphlabels8

ggsave(paste0("ggbiplot_labels_8", ".pdf"), plot = graphlabels8, device = "pdf", path = graphsFolderdirectory, width = 8, height = 6, dpi = 300, units = "in")

dev.new()

group_ellipse8 <- ggbiplot(pca_obj8, ellipse=TRUE, labels = relatdf8$Group_dpi_ID, groups = relatdf8$group)

group_ellipse8

ggsave(paste0("PCA_group_ellipse_8", ".pdf"), plot = group_ellipse8, device = "pdf", path = graphsFolderdirectory, width = 8, height = 6, dpi = 300, units = "in")

summary(pca_obj8) ## shown in **Table S6.**

top_PC1_8

top_PC2_8

**## Principal component analysis: WITH DPI 16 ONLY**

relatdf16 <- relatdf[relatdf$dpi == 16, ]

gene_mat16 <- relatdf16 %>% select(-all_of(nocomp)) %>% as.matrix()

pca_obj16 <- prcomp(gene_mat16, center = TRUE, scale. = TRUE)

summary(pca_obj16)

load_tbl16 <- as_tibble(pca_obj16$rotation, rownames = "gene")

## squared loading × 100 gives % contribution

contrib_tbl16 <- load_tbl16 %>% mutate(across(starts_with("PC"), ~ (.x^2) * 100))

top_PC1_16 <- contrib_tbl16 %>% arrange(desc(PC1)) %>% as.data.frame()

top_PC2_16 <- contrib_tbl16 %>% arrange(desc(PC2)) %>% as.data.frame()

top_PC1_16

top_PC2_16

scores16 <- as_tibble(pca_obj16$x[, 1:2]) # PC1, PC2

relat_pca16 <- bind_cols(relatdf16, scores16)

dev.new()

graphnolabels16 <- ggbiplot::ggbiplot(pca_obj16)

graphnolabels16

ggsave(paste0("ggbiplot_nolabels_16", ".pdf"), plot = graphnolabels16, device = "pdf", path = graphsFolderdirectory, width = 8, height = 6, dpi = 300, units = "in")

dev.new()

graphlabels16 <- ggbiplot(pca_obj16, labels = relatdf16$Group_dpi_ID)

graphlabels16

ggsave(paste0("ggbiplot_labels_16", ".pdf"), plot = graphlabels16, device = "pdf", path = graphsFolderdirectory, width = 8, height = 6, dpi = 300, units = "in")

dev.new()

group_ellipse16 <- ggbiplot(pca_obj16, ellipse=TRUE, labels = relatdf16$Group_dpi_ID, groups = relatdf16$group)

group_ellipse16

ggsave(paste0("PCA_group_ellipse_16", ".pdf"), plot = group_ellipse16, device = "pdf", path = graphsFolderdirectory, width = 8, height = 6, dpi = 300, units = "in")

summary(pca_obj16) ## shown in **Table S6.**

load_tbl16 <- as_tibble(pca_obj16$rotation, rownames = "gene")

contrib_tbl16 <- load_tbl16 %>% mutate(across(starts_with("PC"), ~ (.x^2) * 100))

top_PC1_16 <- contrib_tbl16 %>% arrange(desc(PC1)) %>% as.data.frame()

top_PC2_16 <- contrib_tbl16 %>% arrange(desc(PC2)) %>% as.data.frame()

top_PC1_16

top_PC2_16

**## Principal component analysis: WITH DPI 24 ONLY**

relatdf24 <- relatdf[relatdf$dpi == 24, ]

gene_mat24 <- relatdf24 %>% select(-all_of(nocomp)) %>% as.matrix()

pca_obj24 <- prcomp(gene_mat24, center = TRUE, scale. = TRUE)

summary(pca_obj24)

load_tbl24 <- as_tibble(pca_obj24$rotation, rownames = "gene")

## squared loading × 100 gives % contribution

contrib_tbl24 <- load_tbl24 %>% mutate(across(starts_with("PC"), ~ (.x^2) * 100))

top_PC1_24 <- contrib_tbl24 %>% arrange(desc(PC1)) %>% as.data.frame()

top_PC2_24 <- contrib_tbl24 %>% arrange(desc(PC2)) %>% as.data.frame()

top_PC1_24

top_PC2_24

scores24 <- as_tibble(pca_obj24$x[, 1:2]) # PC1, PC2

relat_pca24 <- bind_cols(relatdf24, scores24)

dev.new()

graphnolabels24 <- ggbiplot::ggbiplot(pca_obj24)

graphnolabels24

ggsave(paste0("ggbiplot_nolabels_24", ".pdf"), plot = graphnolabels24, device = "pdf", path = graphsFolderdirectory, width = 8, height = 6, dpi = 300, units = "in")

dev.new()

graphlabels24 <- ggbiplot(pca_obj24, labels = relatdf24$Group_dpi_ID)

graphlabels24

ggsave(paste0("ggbiplot_labels_24", ".pdf"), plot = graphlabels24, device = "pdf", path = graphsFolderdirectory, width = 8, height = 6, dpi = 300, units = "in")

dev.new()

group_ellipse24 <- ggbiplot(pca_obj24, ellipse=TRUE, labels = relatdf24$Group_dpi_ID, groups = relatdf24$group)

group_ellipse24

ggsave(paste0("PCA_group_ellipse_24", ".pdf"), plot = group_ellipse24, device = "pdf", path = graphsFolderdirectory, width = 8, height = 6, dpi = 300, units = "in")

summary(pca_obj24) ## shown in **Table S6.**
